# Genetic association between gene expression profiles in oligodendrocyte precursor cells and psychiatric disorders

**DOI:** 10.3389/fpsyt.2025.1566155

**Published:** 2025-04-22

**Authors:** Reon Kondo, Hiroki Kimura, Masashi Ikeda

**Affiliations:** Department of Psychiatry, Nagoya University Graduate School of Medicine, Nagoya, Japan

**Keywords:** schizophrenia, autism spectrum disorder, oligodendrocyte precursor cells, single-cell analysis, weighted gene co-expression network analysis

## Abstract

**Background:**

Although neuronal dysfunction has been the focus of many studies on psychiatric disorders, accumulating evidence suggests that white matter abnormalities and oligodendrocyte lineage cells, including oligodendrocyte precursor cells (OPCs), play an important role. Beyond their established contribution to myelination, synaptic genes in OPCs form connections to neurons and influence neuronal circuits and plasticity, thereby potentially contributing to psychiatric pathology.

**Methods:**

We analyzed publicly available single–nucleus RNA sequencing (snRNA–seq) data from white matter cells of healthy donors with SCZ genome–wide association study (GWAS) summary statistics. We assessed cell–type–specific enrichment of SCZ–associated genetic variants and performed weighted gene co–expression network analysis (WGCNA) to identify disease–related gene modules in implicated cell types.

**Results:**

OPCs exhibited significant enrichment of SCZ–associated genetic risk variants and showed pronounced specificity in gene expression patterns. Through WGCNA, we identified a distinct co–expression module in OPCs that was enriched for synaptic genes associated with SCZ.

**Conclusion:**

The present results highlight the previously underappreciated role of OPCs in psychiatric disorders, suggesting that OPC–involved synaptic interactions may contribute to the pathophysiology of SCZ. This work underscores the importance of considering OPCs as active players in neural network dysfunction, with potential implications for future therapeutic strategies.

## 1 Introduction

Psychiatric disorders such as schizophrenia (SCZ) and autism spectrum disorder (ASD) represent a significant public health challenge because of their profound impact on individuals, families, and society (1). These disorders are complex, involving both genetic and environmental factors, and their biological mechanisms remain only partially understood. Recent genomic studies, including genome–wide association studies (GWAS) and analyses of copy number variations and single nucleotide variants, have identified numerous common and rare genetic variants associated with these conditions (2–6). Magnetic resonance imaging (MRI)–based brain imaging studies have revealed altered intra– and inter–regional connectivity, particularly in white matter regions (7–10). However, the specific mechanisms by which genetic variants or alterations in imaging studies contribute to different diseases remain largely unknown.

Advances in single–cell/nucleus RNA sequencing (sc/snRNA–seq) in the postmortem brain have allowed researchers to study the association of genetic variants with different diseases at high resolution, thereby revealing which cell types are most affected by genetic risk variants (11–13). Among these variants, the pathogenetic contributions of neural populations are highlighted, as certain variants affect genes critical for synaptic function, plasticity, and neurotransmission. For example, in SCZ, variants affecting synaptic proteins alter neuronal connectivity (4). Similarly, in ASD, mutations in synaptic genes affect the development of neural circuitry (14).

In addition to direct synaptic interactions between neurons, the role of glial cells, including oligodendrocytes, in neural circuit formation and maintenance, and their contribution to the development of psychiatric disorders, has attracted attention for decades (15–18). Oligodendrocytes are responsible for myelinating axons, thereby enabling rapid electrical signal transmission. Dysregulation of oligodendrocyte function and myelination has been associated with disruptions in both white and gray matter, contributing to impaired synaptic plasticity and altered neural circuits (17). Also, the impairment in the maturation process of oligodendrocyte from their precursor, oligodendrocyte precursors (OPCs) is considered to play a role in the pathogenesis of SCZ (19). MRI studies have revealed significant white matter changes in SCZ and ASD, including reductions in white matter volume and integrity, particularly in brain regions involved in cognition and social behavior (7–10). These white matter abnormalities, observed in both SCZ and ASD, are thought to underlie the neurobiological deficits associated with these conditions.

Recently, weighted gene co–expression network analysis (WGCNA) has been used to elucidate the complexity of psychiatric disorder (20–22). This is a robust approach designed to decipher the intricate relationships within large-scale gene expression datasets. Unlike traditional differential expression analyses, WGCNA constructs networks that reveal clusters—or modules—of highly co-expressed genes, which can then be correlated with clinical traits or experimental conditions such as age, sex, tissue origin, and disease status. This network-based framework transforms gene expression data into a structure where each gene is represented as a node, and the weighted edges signify the strength of the co-expression relationships between gene pairs. By identifying tightly connected gene modules, researchers are able to pinpoint groups that likely function together in specific biological pathways or contribute collectively to phenotypic traits. This holistic methodology not only simplifies the interpretation of high-dimensional data but also facilitates the discovery of key driver genes, or hub genes, that may serve as critical biomarkers or therapeutic targets (23). Moreover, high–dimensional WGCNA (hdWGCNA) ca describe co–expressing gene networks in Alzheimer’s disease and ASD by using single–nucleus gene expression data (24, 25). These methods analyze gene co–expression patterns across single–cell data, revealing cell–type–specific gene modules linked to psychiatric conditions.

Neuronal cell populations have consistently been reported as enriched for the common genetic risk variations linked to psychiatric disorders (4, 11, 26). By contrast, findings regarding OPCs have been more variable (4, 12). Previous studies investigating the association between genetic risk variants and specific cell populations have focused on data derived from gray matter. However, to our knowledge, no studies have examined whether cell populations in white matter are enriched for common genetic risk variations. White matter contains abundant myelinated axons, neurons, and glial cells, including oligodendrocytes and OPCs. White matter microstructural alterations are a shared feature of psychiatric disorders (7), and these pathological changes are not caused solely by abnormal axons. Alterations in white matter neurons and glial cells have also been implicated in the pathophysiology of psychiatric disorders, highlighting the importance of investigating white matter cell populations in relation to genetic risk.

Given this background, we hypothesized that it would be possible to identify novel cell populations and gene clusters implicated in psychiatric disorders by integrating GWAS summary statistics and single–gene expression data, exploring the enrichment of genetic risks in specific cell populations within white matter, and identifying the specific gene modules inside.

## 2 Materials and methods

### 2.1 Study design

**Figure 1:**
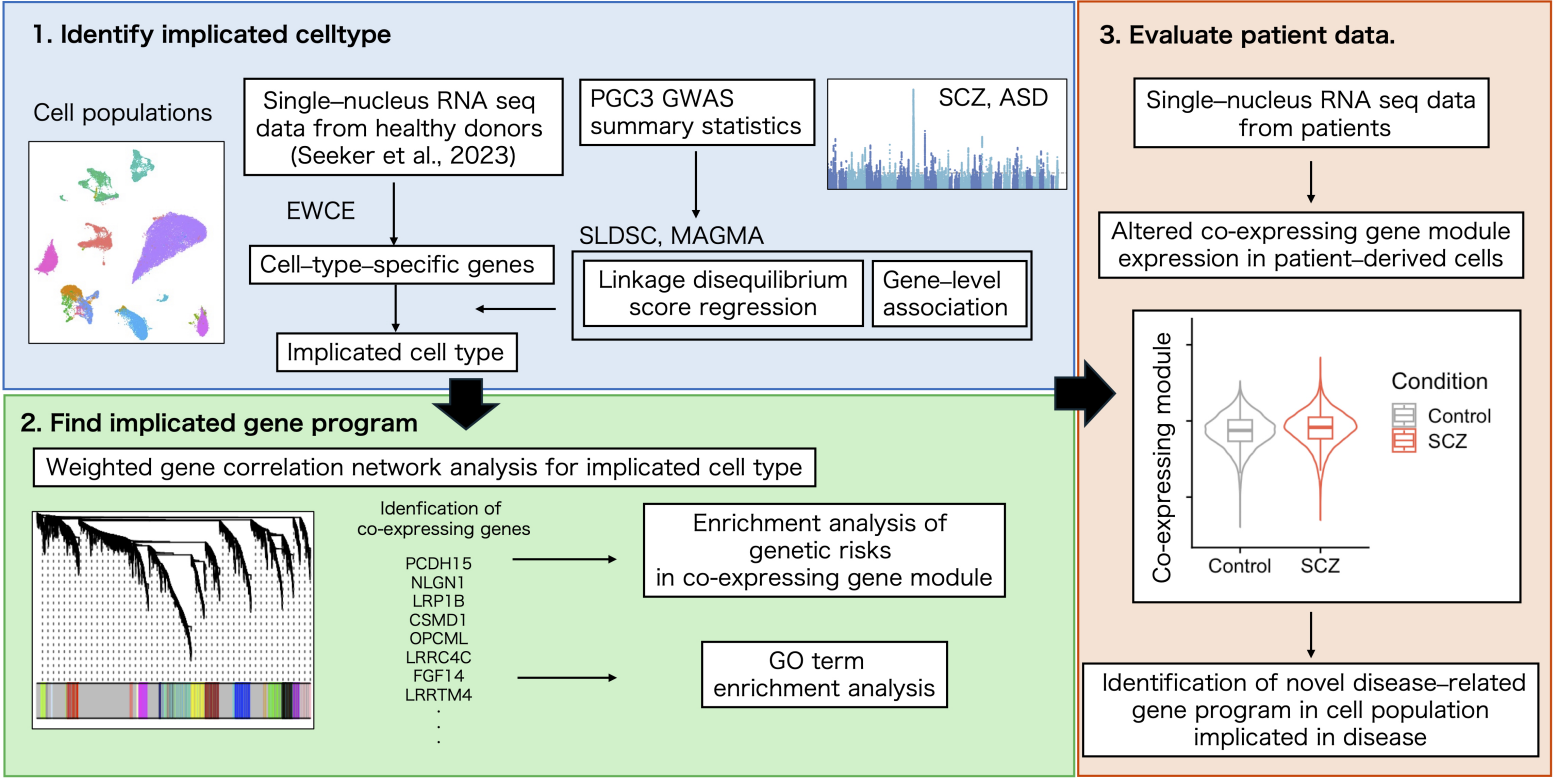
Flowchart of the analysis in this study.

The cell population related to the pathogenesis of SCZ and its functional relevance were explored using summary statistics from GWAS, cluster analysis such as WGCNA and Gene Ontology (GO) term overrepresentation analysis, and single–cell transcriptomic analysis. The study design is shown in **Figure 1**.

### 2.2 Data retrieval

PGC3 GWAS summary statistics were downloaded from the Psychiatric Genome Consortium website. For SCZ GWAS data, summary statistics from the European population were selectively used. This was the largest data set of summary statistics generated from European ethnicity. ASD susceptibility genes were downloaded from the SFARI database (Q1 2024) (27). The single–nucleus gene expression data derived from the white matter of healthy humans with European ethnicity (28) were downloaded from the CZ CELLXGENE Discover database (29), the single–nucleus gene expression data from ASD were downloaded from the UCSC Cell Browser website (30), the single–nucleus gene expression data from patients with SCZ were downloaded from the GEO database under accession number GSE254569 (31), and the bulk RNA seq for OPCs from mouse strain expressing the Disc1–Δ3 gene in OPCs were downloaded from the GEO database under accession number GSE183341 (32).

### 2.3 Preparation of a cell–type–specific gene set

A specificity score was calculated for each gene in each cell population by dividing a gene’s normalized (count per million) unique molecular identifier counts in one cell type by the sum of that gene’s expression in all cell types using the EWCE package (11). Uninformative (i.e., sporadically expressed) genes were removed prior to this process using the *drop_uninformative_genes* function. Genes with a specificity score ranked in the top 10% of the genes expressed were regarded as specific genes in each cell population.

### 2.4 Enrichment of common variant genetic associations using MAGMA

The MAGMA.Celltyping package (12) was used to evaluate the enrichment of common variant genetic associations within each cell population. This package is a wrapper for the gene set enrichment analysis using MAGMA (33). The function *map_snps_to_genes* was run to map single nucleotide polymorphisms (SNPs) in the SCZ GWAS (4) to genes and then to compute gene–wide association *P*–values. The 1000 Genomes data (phase 3) (34) were used as reference to account for linkage disequilibrium (LD) between SNPs. The boundaries of each gene’s transcribed region were extended at a value of 10 kb upstream and 35 kb downstream. This yields, gene–level genetic association for 18,143 genes. Next, a linear regression was run to test for a one–sided association between the top 10% most specific genes in each cell type and the gene–level genetic association with SCZ. Covariates for gene size, gene density, the inverse of the minor allele count, per–gene sample size, and the log of these measures were considered. A total of 15 tests were performed (one for each cell population), and following Cameron et al. (26), we report enrichments with a false discovery rate (FDR) <.05. For the calculation of cell–type–specific gene expression from only BA4 white matter cells, the number of cells in a cell population (central nervous tissue macrophage) was too small (five cells) and thus excluded from the analysis.

### 2.5 Stratified linkage disequilibrium score regression

Following others (4, 12, 26), we also conducted stratified linkage disequilibrium score regression (SLDSR) to assess the enrichment of SCZ SNP heritability in genes in the top expression specificity decile of each cell population. Using HapMap Project phase 3 SNPs with a minor allele frequency >5%, we extended the genomic coordinates for each gene by 100 kb upstream and downstream of the transcribed region, as recommended by the SLDSR authors (13). For each gene set, LD scores were computed for each SNP relative to nearby SNPs within a 1–cM window, utilizing the 1000 Genomes phase 3 reference panel files to estimate LD. SCZ SNP heritability was then stratified for each gene set using a joint fit model accounting for SNP heritability attributable to 53 genomic annotations, including genic, enhancer, and conserved regions (baseline model version 1.2), as performed previously (4, 12, 26). Statistical significance was determined empirically by calculating a *z* score based on whether SCZ SNP heritability was greater in each gene set compared with the baseline model annotations.

### 2.6 Gene set enrichment analysis

GO overrepresentation analyses were performed using clusterProfiler (35). Enriched GO terms were summarized using the rrvgo package (36) to aid the functional interpretation of the enrichment. For the enrichment analysis of common variant genetic associations within genes in each gene module identified in the WGCNA analysis, *calculate_geneset_enrichment* in MAGMA.Celltyping (12) was used. For the gene set enrichment analysis of co–expressing gene modules for ASD susceptible genes in the SFARI database, GeneOverlap (37) was used, with the background regarded as genes that were classified into some of the co–expressing modules. When the gene identification conversion was needed for enrichment analysis, it was performed using *bitr* in clusterProfiler (35).

### 2.7 Weighted gene co–expression network analysis

WGCNA (23) for genes expressed in OPCs was performed using the hdWGCNA package (25) following the authors’ instruction. Briefly, genes to analyze were selected by the criterion of expression in more than 5% of the cells tested. The single–nucleus gene expression data were aggregated into a pseudobulk expression matrix by sample_ids to represent the data from each tissue of each donor in the data set. Pairwise correlations of input features were computed as biweight midcorrelation and weighted with a soft–power threshold of 7. The topological overlap between features was calculated and unsupervised clustering via the Dynamic Tree Cut algorithm (38) was performed to yield a co–expressing gene network. In the identification of module eigen genes, sex, postmortem interval, and age were regressed.

To investigate the module charactericity, differential gene module expression in cell populations, or region of origin, and other features were performed by FindDMEs in hdWGCNA package (25).

For module projection, *ProjectModule*s was executed. The validity of the co–expression network structure in other data sets was evaluated by *ModulePreservation*, which is the implementation of the method by the original WGCNA authors (39). This method calculates several network–related metrics and summarizes the features into a Z summary. The resulting Z summary value for preservation was used for the evaluation, and values above 10 were regarded as significant.

### 2.8 Alterations in the gene set expression of OPCs in different pathological conditions

The cell population labels were adopted from an original analysis of each data set. The data from cells whose cell–type label corresponded to OPCs were extracted from each data set and the module scores for the SCZ–implicated gene modules were computed by *Addmodulescore* in Seurat (40). For SCZ, 20,947 nuclei from the orbitofrontal cortices of 36 patients were compared with 16,481 cells from 33 controls (31). For ASD, 5,340 cells from the prefrontal cortices of 13 patients were compared with 4783 cells from 10 controls (30). The statistics were first performed by comparing nuclei–wise module scores. Then, for individual level statistics, module score was averaged for nuclei from the same donor. Sex, age, RNA integrity number (RIN), post–morten interval (PMI) and Brain.pH were regarded as covariates (for the ASD dataset, Brain.pH was not available). The formula was disease_status ~ averaged module score + sex + age + RIN + PMI + Brain.pH + (1| lib_batch), and statistics were calculated using lmerTest (41).

The differentially expressing genes (DEGs) for bulk RNA–seq from murine OPCs expressing Disc1–δ3 were computed using DESeq2 (42). *P*–values were adjusted for multiple testing using the FDR method considering all tested genes. The DEGs with an adjusted *P*–value of < 0.01 were considered for the downstream analysis. The statistics were computed using Fisher’s exact test, and the resulting *P*–values were adjusted using the Benjamini–Hochberg procedure.

### 2.9 Pseudotime trajectory analysis

Pseudotime trajectory analysis of white matter oligodendrocyte lineage cells was performed using Monocle3 according to the authors’ instructions (43). Briefly, clustering was performed at a resolution of 1e–4 using cluster function. The principal node within the OPC cluster located at the opposite side of the differentiation–committed OPC cluster was used as a starting point, and the pseudotime was calculated. Module gene set expression along the trajectory was performed based on hdWGCNA documentation.

### 2.10 Compositional analysis

Compositional differences between patients and controls for the SCZ dataset were calculated using cacoa (44). The algorithm was used in a previous study (45). The cluster–free compositional difference was investigated for OPCs from the data set. Briefly, at first, densities for each sample are calculated using either a 2D embedding (via kernel density estimation, where the space is divided into a 400×400 grid and normalized). Next, The difference in densities between conditions is computed for each data point using the Wilcoxon test statistic, which was chosen for its power. To assess significance, condition labels are permuted 400 times, and the entire procedure is repeated. A permutation–based p–value correction method was then applied to adjust for multiple comparisons.

### 2.11 Statistical analysis

All analyses except for SLDSC analysis were performed using R version 4.3.4 (46) and R studio (2024.09.1 + 394) (47). The manipulation of single–nucleus gene expression data was performed using Seurat 5.0.1 (40). The Wilcoxon rank–sum test was used to compare the measurement data. Gene set enrichment was tested using a one–tailed Fisher’s exact test. *P*–values < 0.05 were considered statistically significant.

## 3 Results

### 3.1 Oligodendrocyte precursor cells mediate certain psychiatric genetic risks

We tested whether cell populations in white matter mediate the common genetic liability to SCZ using publicly available single–nucleus data obtained from the white matter of healthy subjects of European ethnicity. The single–nucleus data for white matter were composed of 45,852 cells classified into 15 cell types based on the analysis in the original study and the CELL×GENE database. For easier interpretation of the data, we renamed two of the cell populations labeled in the data set as ‘cerebellar granule cell’ and ‘neuron’ because these populations are not restricted to the cerebellum and express the marker gene CRH (**Supplementary Figure 1**).

**Figure 2:**
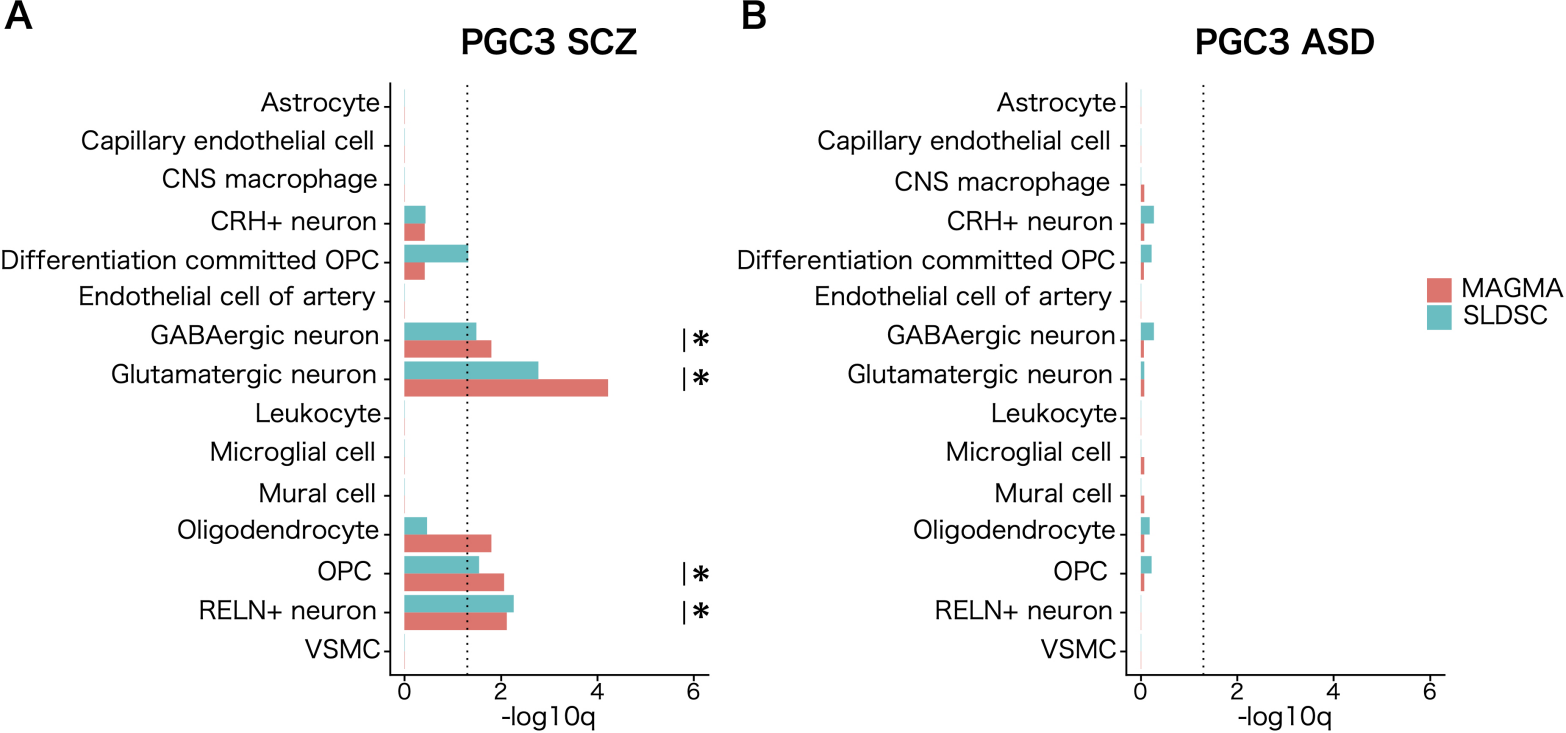
Cell–type–specific enrichment of common genetic risk for psychiatric disorders in white matter. Cell–type–specific enrichment of common genetic associations for **(A)** schizophrenia (SCZ) and **(B)** autism spectrum disorder (ASD) calculated for each cell type using MAGMA (red) and SLDSC (blue). The genes in the top expression specificity deciles of each cell population were regarded as cell–type–specific genes. The dotted line shows the significance threshold (q <.05). Asterisks indicate cell populations that satisfied FDR <.05 in both S–LDSC and MAGMA analyses.

To implicate the cell population in white matter that mediate the common genetic liability to SCZ, we calculated the top decile of gene expression specificity for each of the labeled cell populations and used the statistical methods MAGMA (12, 33) and SLDSR (13) to assess SCZ genetic risk enrichment. Both analyses showed significant enrichment (FDR <.05) of SCZ genetic risk in genes with high expression specificity for three neuronal cell populations and OPCs. For oligodendrocytes, significant enrichment was only observed for MAGMA analysis and not for SLDSR analysis. Likewise, cell–type–specific enrichment was tested for associations with common variants with ASD, and no enrichment was observed in OPCs (**Figure 2**; **Supplementary Table 1**). The enrichment signal in OPCs held when tested with 9,975 cells derived from cortical white matter (**Supplementary Figure 2**). When cell–type–specific enrichment was tested for detailed 60 subcluster cell types annotated in a previous study (28), no significant enrichments were observed for 11 subpopulations in the oligodendrocyte lineage (data not shown).

We also tested the enrichment of 1,176 ASD susceptibility genes reported in the SFARI database, including rare single–gene mutations. Using the EWCE package, we assessed enrichment for the top decile of gene expression specificity for each cell population. The analysis yielded statistically significant enrichment of strong candidate ASD susceptibility genes in glutamatergic neurons and OPCs (**Supplementary Figure 2**). This result suggested disruption of the ASD susceptibility genes involved in the pathogenesis of ASD through their functions in OPCs in white matter.

Next, we explored the extent to which observed enrichments of genetic associations and susceptibility genes in different populations of white matter cells represent independent signals. Overlap of 5,604 nominally significant SCZ–associated genes (MAGMA gene–wise *P* <.05) was observed within the top expression specificity decile of the four cell populations of the white matter implicated in the disorder. Our analysis revealed partially overlapping enrichment signals among these populations.

**Figure 3:**
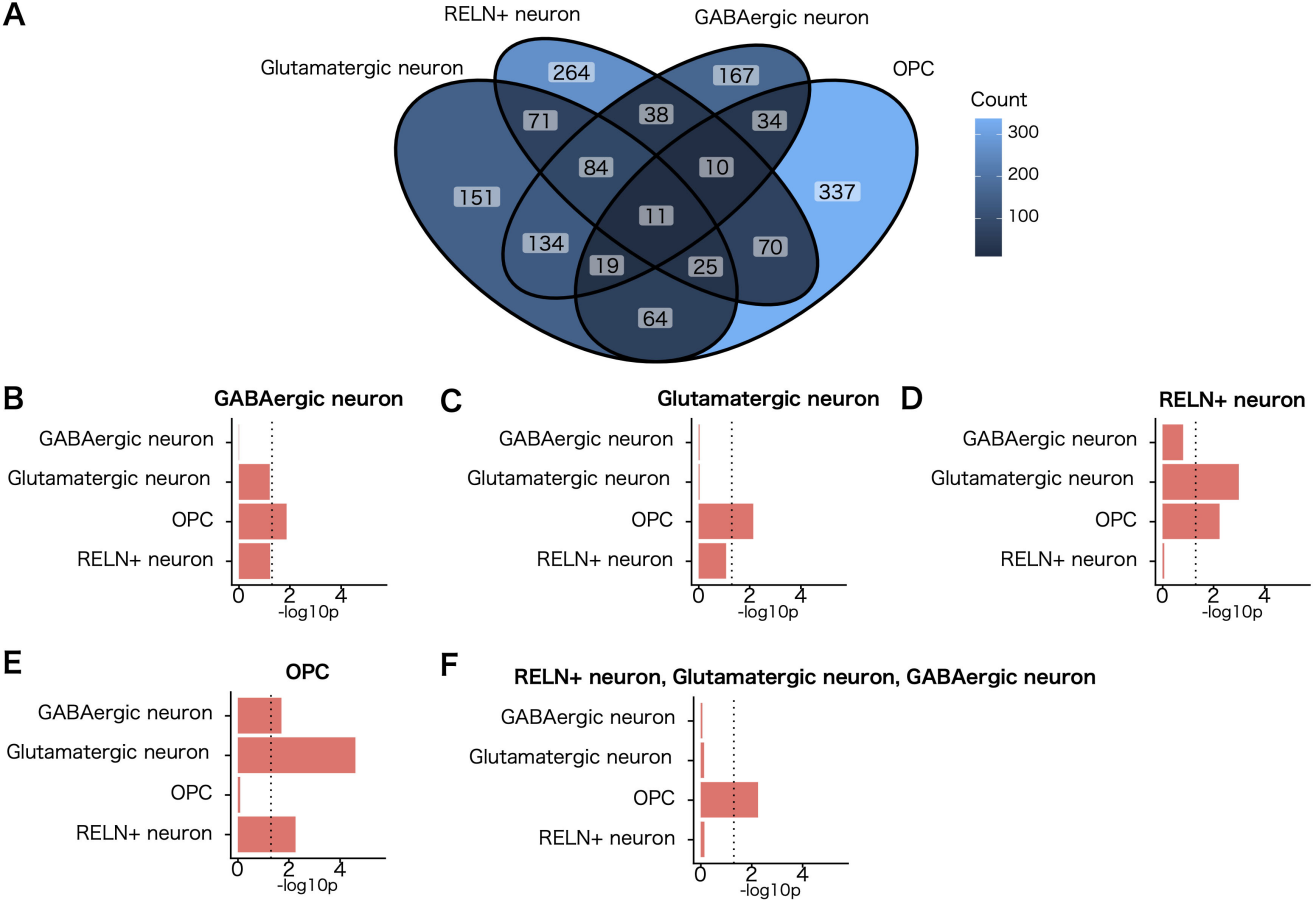
Evaluation of independent cell–type–specific enrichment signals for SCZ. **(A)** Venn diagram of the number of genes exhibiting a nominally significant genetic association with SCZ (MAGMA gene–wise *P* <.05) shared between the top expression specificity deciles of each cell population implicated in the disorder at a level exceeding an FDR <.05 threshold in both MAGMA and stratified linkage disequilibrium score regression analysis. **(B–F)** MAGMA cell–type–specific gene–enrichment analysis of common schizophrenic genetic associations controlled for each cell population implicated in the disorder. The cell populations controlled for are shown above each bar graph. Dotted lines indicate the nominal significance threshold (*P* = .05).

To determine whether the enrichment of SCZ genetic associations in OPCs is independent of genes shared with enriched neuronal cell types, we repeated the MAGMA cell–specific expression analyses for other implicated cell types while conditioning genes in the top expression specificity decile of the implicated neuronal cell types (**Figure 3A**). Notably, the OPC cell population remained significantly enriched for SCZ genetic associations (*P* < 0.05) after accounting for genes shared with neuronal cell types (**Figures 3B–F**). These findings indicate that the enrichment of SCZ genetic associations in OPCs represents signals independent of neuronal contributions. Although OPCs only meet the Bonferroni threshold in MAGMA analysis and the FDR threshold in SLDSC analysis, their physiological roles related to myelination, coupled with the independence of their genetic association signals, warranted their prioritization in subsequent analyses.

### 3.2 Functional enrichment of the top gene expression specificity decile of OPCs

To elucidate the cellular processes associated with genes in the top decile of gene expression specificity for each cell population, we performed GO term overrepresentation analysis using clusterProfiler (35). The findings revealed significant enrichment of synaptic gene expression in OPCs (**Supplementary Figure 3A).** Semantic similarity–based summarization of the enriched terms revealed that synapse–related terms were the most prominently enriched (**Supplementary Figure 3B**). Furthermore, the overlaps between nominally significant SCZ–associated genes, SFARI ASD risk genes, and OPC specific genes in white matter were enriched for synaptic genes (**Supplementary, Figure 3C**). These findings suggest that synaptic gene function in white matter OPCs plays a critical role in the white matter alterations observed in the disorder.

### 3.3 WGCNA analysis of white matter OPCs

**Figure 4:**
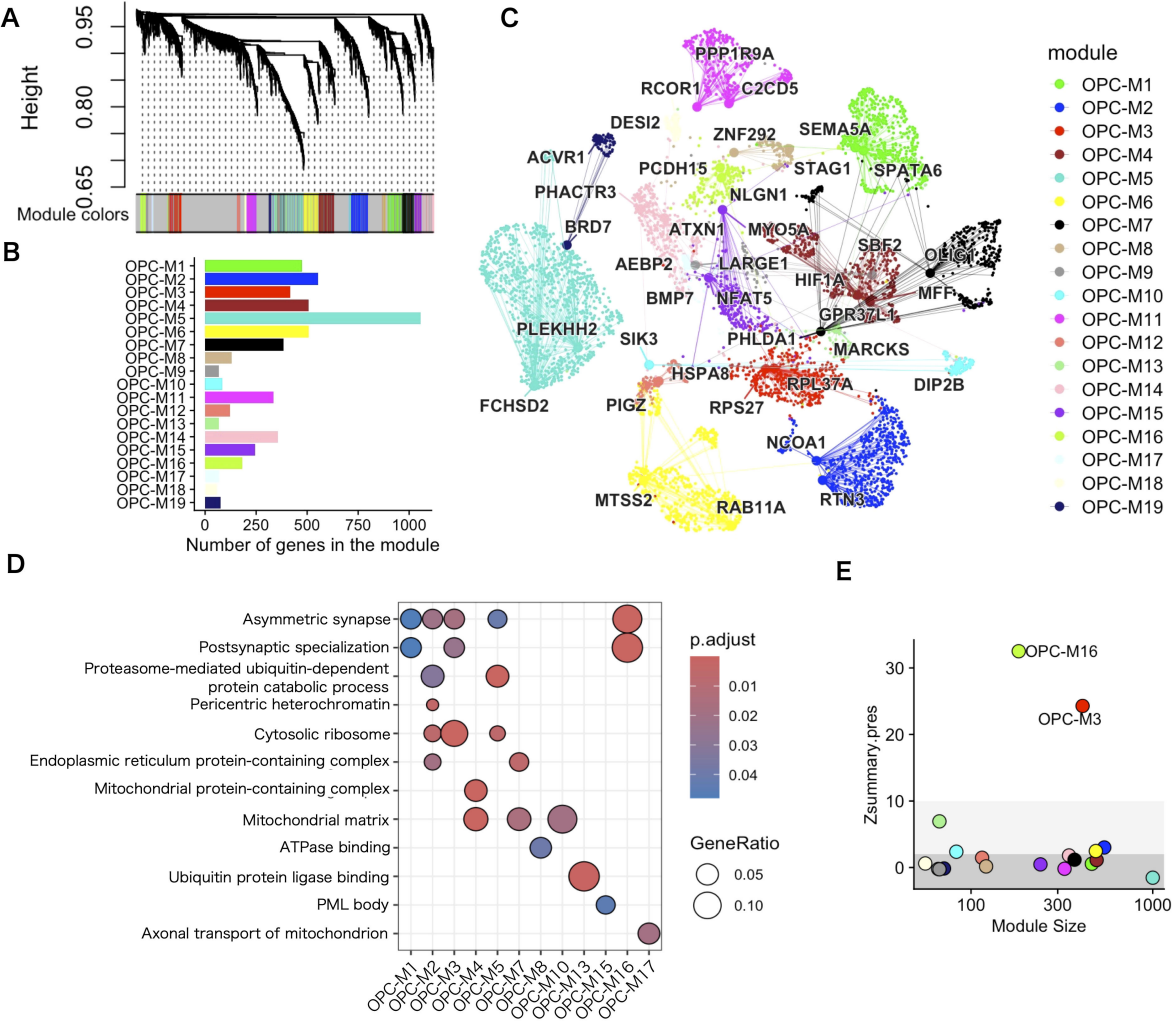
WGCNA analysis of white matter OPCs identified in several co–expressing gene modules. **(A)** Hierarchical clustering dendrogram of a co–expressing gene network. **(B)** Number of genes in each co–expressing gene module. **(C)** UMAP plot of the co–expression network. Each node represents a single gene, and edges represent co–expression links between genes and module hub genes. Nodes are colored according to their co–expression module assignment. The top two hub genes per module are labeled. Network edges were down–sampled for visual clarity. **(D)** GO term overrepresentation analysis for each co–expressing gene module. For visibility, only the top pathways enriched in any of the co–expressing modules are shown. Statistics were performed using Fisher’s exact test and only GO terms with a *P*–adj <.05 were considered significantly enriched. **(E)** The module preservation score of each colored dot represents the score for each co–expressing module. The thick gray area (Z < 2) indicates that the co–expressing module is not preserved, the pale gray area (2 < Z ≤ 10) that co–expressing module is moderately preserved, and the white area (Z > 10) that the co–expressing module is highly preserved.

To investigate further the gene program in which genetic risks influence OPCs, we applied WGCNA to the 3094 OPCs in the white matter single–nucleus RNA–seq data. The pipeline implemented in hdWGCNA (25) was used to create a pseudobulk expression matrix for each of the biological replicates, (i.e., each region from each donors), in the data set and construct a co–expression gene network. We applied pseudobulk analysis because the numbers of OPCs were small in each biological replicate of the white matter data set. To highlight the strong positive correlations, co–expression similarity was transformed into a signed and weighted adjacency matrix by a soft–thresholding method that yielded approximate scale–free topology. Then, the resulting adjacency matrix was converted into topological overlap measures (TOMs) and hierarchical clustering via the Dynamic Tree Cut algorithm (38) was performed to yield 19 co–expression modules (named sequentially OPC–M1 to M19 without consideration for functional relevance), from 9768 genes based on the gene expression profile in OPCs (**Figures 4A, B**). Subsequently, module eigengenes (MEs), defined as the first principal component of the module’s gene expression, were calculated to represent the expression of each co–expression module in OPCs. The genes classified into co–expression modules were ranked by similarity of their network connection with other genes to that of MEs (kMEs). To visualize the co–expressing gene network, uniform manifold approximation and projection (UMAP) was applied to embed the TOM co–expression network into a two–dimensional manifold using the topological overlap of each gene with the top 25 genes from each module as input features (**Figure 4C**). To visualize the module specificity in OPCs and other cell populations, the module was back–projected to the entire dataset. OPC–M16 was the most significant differentially expressing module in OPCs (**Supplementary Figure 5**). To annotate the identified co–expression modules functionally, an overrepresentation analysis of GO terms was performed. The results showed that at least one GO term was overrepresented in 12 co–expression modules (**Figure 4D**). Remarkably, some of the co–expression modules showed enrichment of synaptic genes, with OPC–M16 being the most enriched module. When the effect of sex was tested, no co-expression modules showed significant difference for sex. As for regional difference, OPC-M16 was most prominently expressed in OPCs from cortical white matter (**Supplementary Figure 6**).

To test the validity of the co–expressing module in the other data sets, we evaluated co–expressing network preservation. First, we tested whether the co–expressing module was limited to the input white matter data and found that the single–nucleus data of 105,734 OPC nuclei from 107 brain areas containing gray and white matter (48), to find the existence of a similar network structure in terms of genes in some of the co–expressing gene modules. The OPC–M3 and OPC–M16 modules were highly preserved (Z > 10) in the data set (**Figure 4E**), which suggests that some of the gene co–expressing modules are common features of OPCs, regardless of the region from which they are derived.

### 3.4 Enrichment of genetic risk in specific co–expressing modules in OPCs

**Figure 5:**
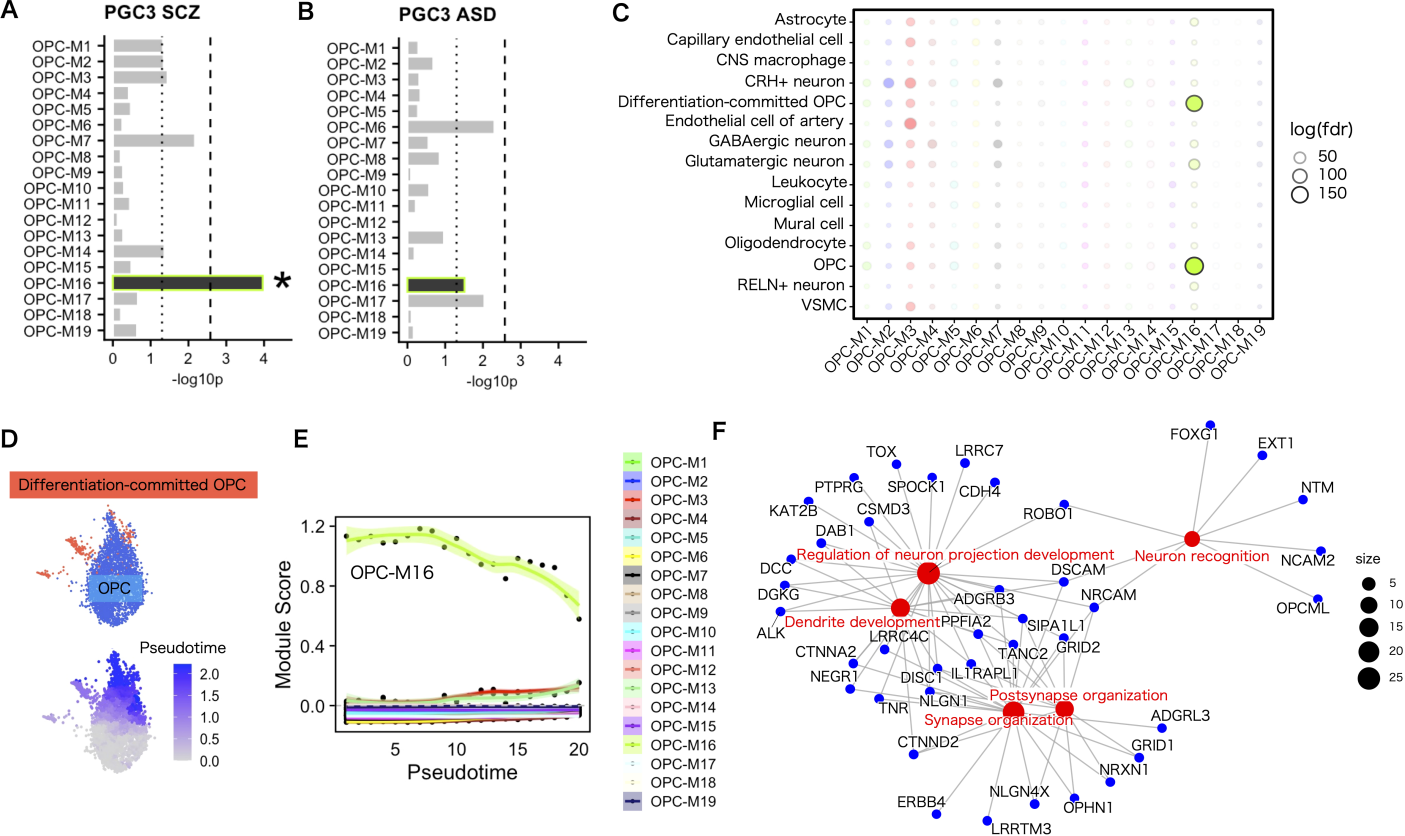
Enrichment of genetic risks for SCZ in a specific gene module in OPCs. **(A, B)** Gene set enrichment analysis for common genetic associations using MAGMA. The genetic risks for SCZ were implicated in a co–expressing gene module in OPCs **(A)**, whereas in ASD, no significant implication was observed **(B)**. Dotted lines indicate the nominal significance threshold (<.05). Asterisks indicate modules that satisfied Bonferroni–corrected significance (*P* <. 2.6×10^–3^). **(C)** Overlap of a co–expressing gene module in OPCs and cell–type marker genes. Statistics were performed using Fisher’s exact test. Marker genes were defined by *FindAllMarkers* in Seurat with p < 1.0×10^–4^ and a log–fold change > 1. **(D)** OPC and differentiation–committed OPC cell–type labels in the data set (upper). Pseudotime trajectory from OPCs to differentiation–committed OPCs (lower). **(E)** Expression level of OPC–M16 along the pseudotime trajectory from immature to differentiation–committed OPCs. Pseudotimes were shown in 20 binned categories. Each dot represents the average module score for each co–expressing module in each pseudotime bin. **(F)** Concept network plot of genes in the top five enriched GO terms for OPC–M16. Statistics were performed using Fisher’s exact test and *P*–adj <.05 was considered significant enrichment.

Co–expressing gene module–specific enrichment analysis of psychiatric genetic risk associations was performed using MAGMA. Surprisingly, the common genetic risks for SCZ were enriched with Bonferroni–corrected significance (p < 2.6×10^–3^) in a single co–expression module (OPC–M16) that showed the most prominent representation of synaptic proteins (**Figures 5A, F**). Common genetic risks for ASD did not meet the level of significance on the co–expressing module (**Figure 5B**). The EWCE enrichment analysis of ASD susceptibility genes from SFARI revealed enrichment of risk genes in the same single co–expressing gene module (**Supplementary Figure 7**).

Because synaptic genes are also expressed in neuronal cell types, we validated the co–expressing gene module specificity for each cell type. Overlap analysis of co–expressing gene modules and cell–type markers revealed that OPC–M16 showed the largest overlap with markers for OPCs and their differentiated cells, differentiation–committed OPCs (**Figure 5C**). On the other hand, the gene module showed reduced overlap in oligodendrocytes. Thus, among the oligodendrocyte lineage cell types, the expression level of OPC–M16 was highest in OPCs, medium in differentiation–committed OPCs, and lowest in oligodendrocytes, suggesting their function along the differentiation process (**Supplementary Figure 4**). Therefore, we attempted pseudotime trajectory analysis from OPCs into differentiation–committed OPCs and found the expression level of the gene module decreased along the pseudotime trajectory supportive of its involvement in the differentiation process (**Figures 5D, E**).

The genes in the disease risk gene–enriched module (OPC–M16) in the top five enriched GO biological process terms are listed in **Figure 5F** (the top 10 genes ranked by kME values are listed in **Supplementary Table 1**). These contain several known canonical OPC and synaptic genes, among which, PCDH15 stands out for its established role in OPC proliferation and morphological development, as well as its use as a reliable OPC marker in single–cell transcriptomic analyses (49). Notably, DISC1 is a gene expressed within the oligodendrocyte lineage, with experimental evidence confirming their involvement in the oligodendrocyte maturation (50). DISC1 is also involved in synaptic structure and the coded protein interacts with many of the synaptic proteins (51). Other synaptic genes such as NLGN1 and GRIK2 play essential roles in glutamatergic neurotransmission, with NLGN1 being part of the neuroligin family of postsynaptic adhesion proteins that modulate synaptic plasticity and cognitive function (52). GRIK2, a glutamate receptor, is critical in excitatory synaptic signaling. NRXN1, a presynaptic protein, interacts with neuroligins (including NLGN1) at synapses, forming a structural and functional bridge between pre– and postsynaptic sites (52). Although the precise function of many of these and other genes in OPCs remains poorly understood, together, these genes offer insights into the molecular underpinnings of both OPC development and synaptic architecture, further suggesting their relevance in understanding the complex interplay between myelination and synaptic transmission in psychiatric disorders.

### 3.5 OPC co–expressing modules in the data set derived from pathological conditions

**Figure 6:**
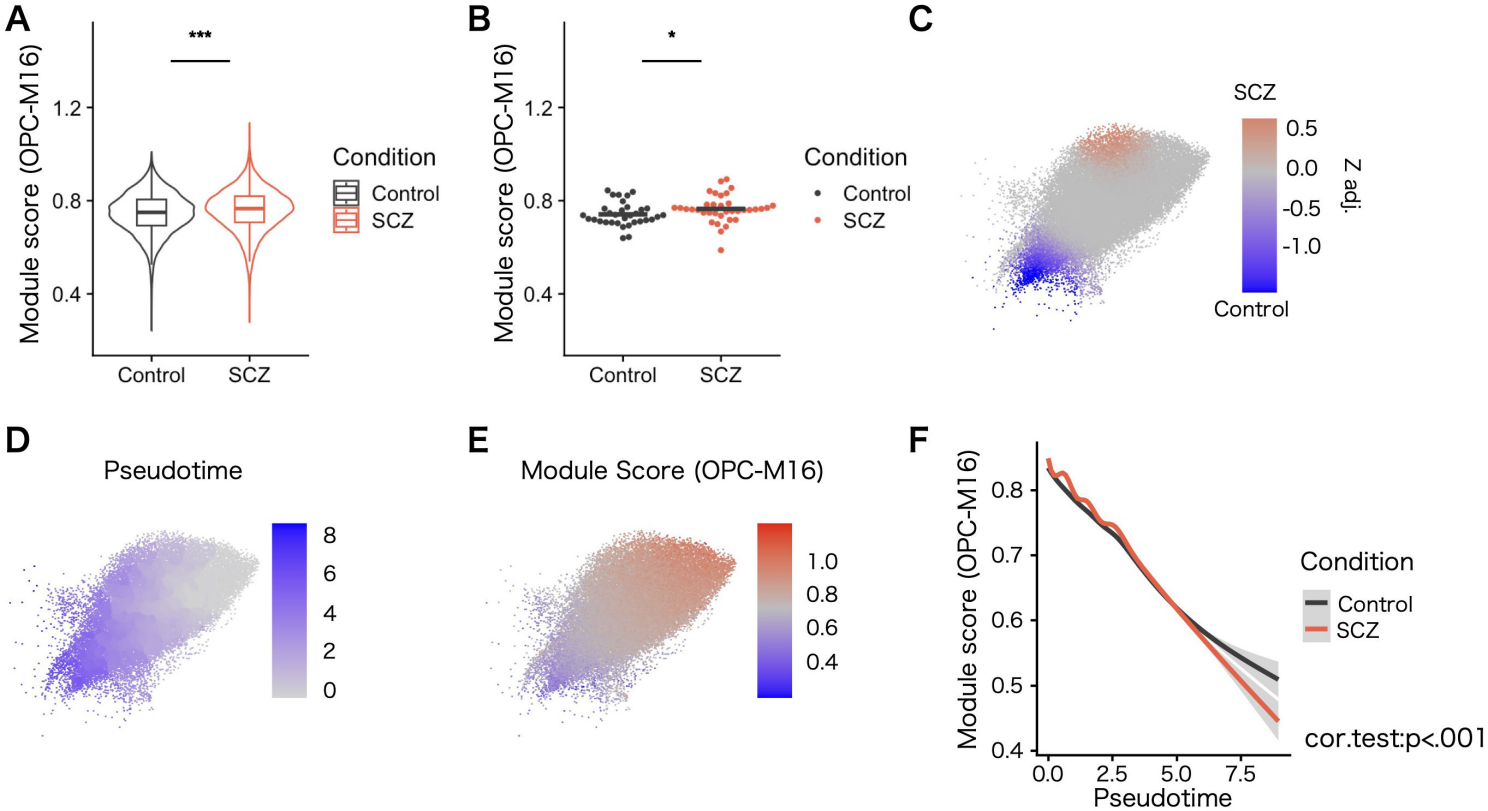
Expression of the co–expression gene module OPC–M16 in the SCZ patient–derived data set. **(A)** Expression level of OPC–M16 calculated as a module score. Compared with controls, patients with SCZ exhibited signi cantly elevated OPC–M16 module scores in OPCs. Statistical signi cance was determined using the rank–sum test. Error bars represent the standard error of the mean. **(B)** The elevation remained signi cant in patients with SCZ when tested by averaging cells from the same patients and after adjusting for covariates. Statistical signi cance was determined using linear mixed–effects models, accounting for relevant covariates. **(C)** Case–control compositional analysis using OPCs from the SCZ data set. Z–values and areas with statistical signi cance (FDR <.05) with adjusted z–values are shown. Red indicates more cells from patients with SCZ, and blue indicates more cells from controls. **(D)** Pseudotime trajectory analysis based on the expression levels of OPC markers not included in OPC–M16. **(E)** Heatmap of OPC–M16 module scores. Values were scaled in FeaturePlot in Seurat. **(F)** Correlation between pseudotime and OPC–M16 module scores. Smoothed lines for each condition are plotted and gray areas show 95% con dence intervals. For each condition, the progression of pseudotime and decreased module scores for OPC–M16 were correlated (*P* <.001). For visibility, OPCs that were within 5 SD of the mean UMAP coordinates are shown (20 cells that are outside were excluded from the plot above). *P < 0.05, ***P < 0.001.

We explored the expression level of co–expressing modules in different pathological conditions. Given that current publicly available single-nucleus gene expression data for SCZ and ASD are limited to cortical gray matter nuclei, we evaluated the expression of the disease risk module by calculating the module score in OPCs from the gray matter of controls and psychiatric patients in each dataset (SCZ and ASD). The nuclei–wise comparison and individual-level comparison accounted for covariates indicated altered expression of the module (OPC–M16) in OPCs from patients with SCZ (**Figures 6A, B**; **Supplementary Figure 8**). The co–expressing network structure related to OPC–M16 was highly preserved in data sets including patinet–derived nuclei and warrants the comparability (**Supplementary Figure 9**).

To investigate alterations in OPCs from patients with SCZ that might contribute to the pathophysiology of the disease, compositional analysis (44, 45) was performed to find subpopulational change. The analysis revealed a significant difference in the composition of OPCs (**Figure 6C**). Comparing the expression of known differentiation markers of oligodendrocyte lineage could show that the subpopulational difference might correlate with the differentiation process. When pseudotime trajectory analysis was applied based on the expression of oligodendrocyte lineage markers not included in OPC–M16 (**Supplementary Figure 10**), more abundant immature state OPCs were found in SCZ (**Figures 6C, D**). In addition, the differentiation process was correlated by OPC–M16 genes, which was enriched for synaptic genes (**Figures 6E, F**). These results suggest the presence of transcriptional changes also in white matter OPCs and impairment in the differentiation process of OPCs in SCZ patients.

## 4 Discussion

In this study, we explored the cell–type–specific genetic risk enrichment of psychiatric disorders within white matter tissue and identified OPCs as a carrier of SCZ–associated genetic risks related to myelination. We also found that OPCs in white matter exhibit enriched expression of ASD susceptibility genes. WGCNA analysis further revealed significant enrichment of psychiatric disorder–associated genes within a co–expressing gene module in OPCs. Pathway analysis indicated that the effect of these genetic variants converges on synaptic genes expressed in OPCs. In line with this, the expression levels of the co–expressing genes were altered in OPCs derived from patients with SCZ, suggesting that disruptions in the function of synaptic genes of OPCs contribute, at least partially, to the neurobiological mechanisms underlying psychiatric disorders.

As their name signifies, OPCs are classically known for their ability to differentiate into oligodendrocytes and contribute to myelination. However, recent studies have depicted their wide variety of physiological functions. For example, they are involved with the migration of neuronal cells (53), axonal regeneration upon injury (54), engulfment of synapses to sculpt neural circuits (55), and interaction with vessels (56, 57). Regarding myelination, the proliferation and differentiation of OPCs are meticulously regulated in a neural activity–dependent manner and not random. They can make synaptic contacts with neurons and receive electrical signals from neurons (58). Experimentally, the role of synapse and synaptic genes in OPCs in their differentiation has been reported (59). Moreover, impaired differentiation of OPCs has been reported in patients with SCZ (18), and oligodendrocyte maturation has been suspected as a biological mechanism underlying the pathogenesis of SCZ (19). These findings suggest a relationship between synaptic gene expression in OPCs and their differentiation, along with the resulting myelination of axons. Supporting our results and speculations, hyperbranching morphology, which is suggestive of altered connections with other OPC cells in patients with SCZ, has been reported (32). A similar hyperbranching morphological phenotype has been observed in OPCs derived from conditional knockout model mice with Disc1–δ3 that lacks functional Disc1 specifically in OPCs, one of the gene in OPC-M16 (32). In that model, we performed differential expression analysis and GO term enrichment analysis to confirm the presence of elevated synaptic gene expression levels in OPCs (**Supplementary Figure 11**), and the original analysis revealed the OPC phenotype causes a loss of synapses in neurons, together indicating a causal association between a gene belonging to OPC-M16 genes in OPCs and neural circuits in the pathogenesis of the disease. Regarding ASD, other studies have suggested that synaptic connection signals, including NRXN1–NLGN3, to OPCs are reduced in such patients (60), highlighting the potential role of OPC synaptic gene alterations in the communication between OPCs and other cells.

Recent studies have reported sex differences in OPC characteristics. For example, *in vitro* studies indicate that female OPCs exhibit a higher capacity for proliferation and migration, whereas male OPCs tend to differentiate and myelinate more effectively (61). Clinically, schizophrenia also displays sexual dimorphism: male patients often experience an earlier onset, more severe negative symptoms, greater white matter disruptions, and significant oligodendrocyte loss, while female patients generally have a later onset and milder early symptoms with distinct affective and cognitive profiles (62–64). In ASD, males typically show more pronounced symptom profiles and network hypoconnectivity, whereas females exhibit network hyperconnectivity Although few studies have directly linked OPC differences to sex-specific aspects of SCZ or ASD, future research integrating clinical, histopathological, imaging, and transcriptomic data could yield important insights into these variations.

While this study presents promising insights, certain limitations highlight areas for future exploration. One key limitation is the limited availability of white matter data. Thus, although the network structure of the co–expressing genes was preserved in the data from other brain regions, a direct investigation of white matter data from patients is needed. Furthermore, the data set containing white matter and gray matter from same donors that enables evaluation of the effect of individual genetic variation to the regions and inter-region relationships would greatly enhance the understanding of white matter alterations. The intricate interaction between gray and white matter could not be evaluated by currently available data. Because axons myelinated in white matter predominantly originate from neuronal cell populations in gray matter, genetic risks that affect neurons in gray matter also influence cells in white matter. This interrelationship needs to be considered to gain a more complete understanding of the precise biological mechanisms underlying the pathogenesis of the disease. In addition, psychiatric genetic risks, particularly those related to synaptic function, exert their effects through not only OPCs, but also neuronal cell populations. Importantly, these genetic risks can impact patient life–spans from prenatal development to senescence. This temporal dimension necessitates a more comprehensive understanding of the development of the nervous system and the aging process. Thus, while the precise magnitude of the effects mediated by OPCs remains challenging to estimate with current data, ongoing advances in data availability, multimodal integration, and analytical techniques provide a promising path forward. By addressing these gaps, future research holds the potential to unlock deeper insights into the role of OPCs and broader neural networks in psychiatric disorders, ultimately guiding the development of novel therapeutic approaches.

## 5 Conclusion

This study highlights the potential contributions of synaptic genes in OPCs to the pathogenesis of psychiatric disorders, especially SCZ and ASD. Further validation using larger data sets from human patients, along with the integration of data from other modalities (e.g., spatial transcriptomics, single–nucleus ATAC–seq, individual–level genomic data), is therefore essential. Additionally, elucidating the precise molecular mechanisms by which OPCs mediate psychiatric genetic risks in pathophysiology and pathological development could offer novel perspectives and potential therapeutics for psychiatric disorders.

## Data Availability

Publicly available datasets were analyzed in this study. This data can be found here: <b>Links for the data used were followings:CZ CELLXGENE Discover database (white matter single cell data): https://cellxgene.cziscience.com/e/c05e6940–729c–47bd–a2a6–6ce3730c4919.cxg/, GEO database (human SCZ single cell data): https://www.ncbi.nlm.nih.gov/geo/query/acc.cgi?acc=GSE254569, GEO database (murine SCZ model bulk RNA–seq data): https://www.ncbi.nlm.nih.gov/geo/query/acc.cgi?acc=GSE183341, PGC3 GWAS summary statistics: https://pgc.unc.edu/for–researchers/download–results/, SFARI GENE (accessed in Augst 2024): https://gene.sfari.org, UCSC Genome Browser (human ASD single cell data): https://cells.ucsc.edu/?ds=autism .
