# Supplementary material for: Genetic association between gene expression profiles in oligodendrocyte precursor cells and psychiatric disorders
Source: Front Psychiatry. 2025 Apr 22;16:1566155. doi: 10.3389/fpsyt.2025.1566155 (PMC12054250; doi:10.3389/fpsyt.2025.1566155)
Supplement: Supplementary file 1 [file SupplementaryFile1.docx]

Supplementary Material

# Supplementary Figures and Tables

## Supplementary Figures


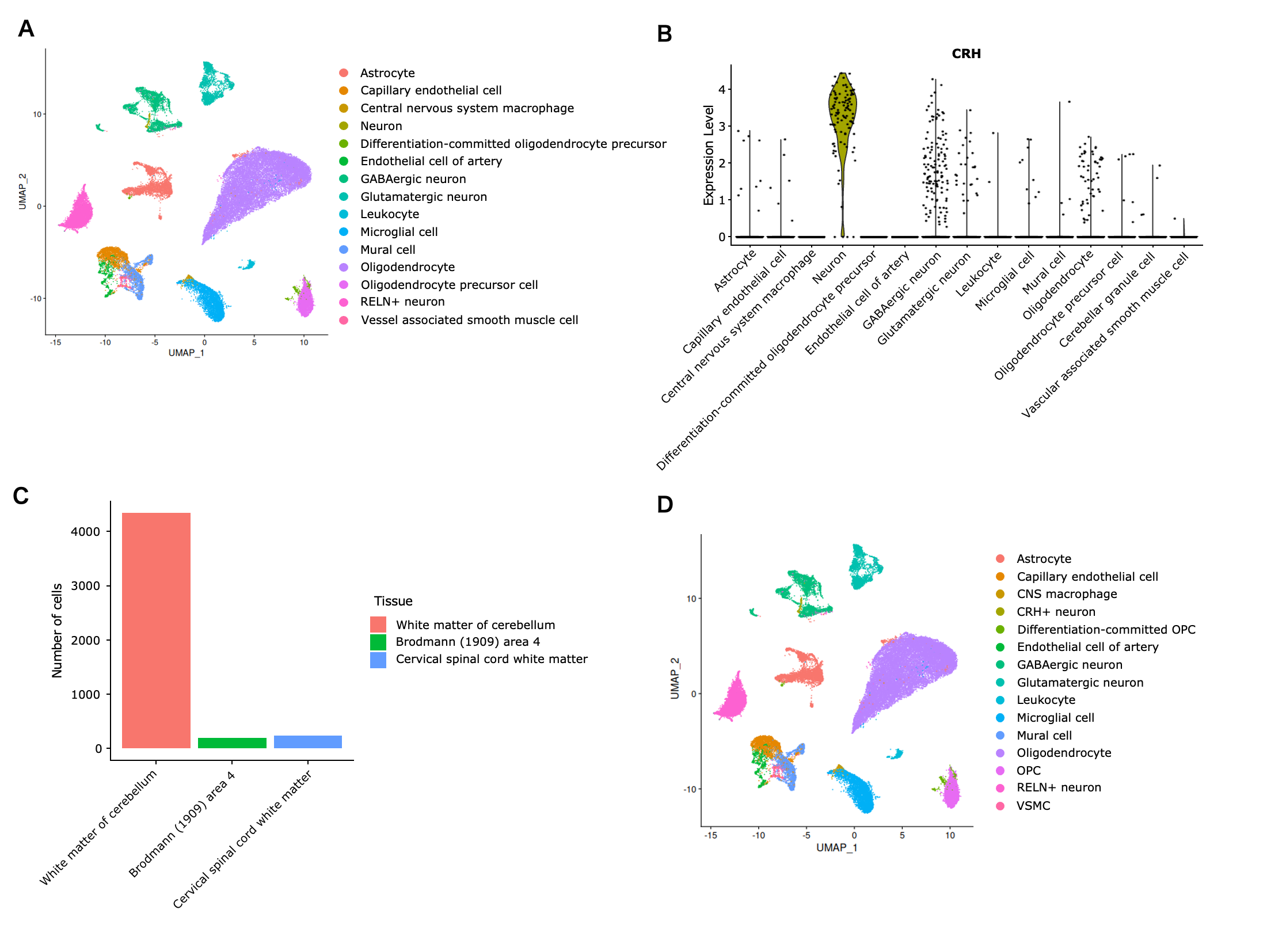


**Supplementary Figure 1.** Modification of some labels in the data set. (A) UMAP coordinates and cell-type classification of the data set downloaded from CELLXGENE. (B) CRH expression levels in each cell-type population. The cell population labeled as ‘Neuron’ in the CELLXGENE data set highly expressed CRH transcripts with high specificity. (C) Number of cells in a cell population labeled cerebellar granule cells in each region. Not only cerebellar, but also cortical and spinal white matter, harbor this cell population. (D) Renamed cell populations. Some names are shown as abbreviated name. CNS: central nervous system, CRH: corticotropin-releasing hormone, GABA: gamma amino butyric acid, OPC: oligodendrocyte precursor cell, RELN: reelin, VSMC: vascular-associated smooth muscle cell.


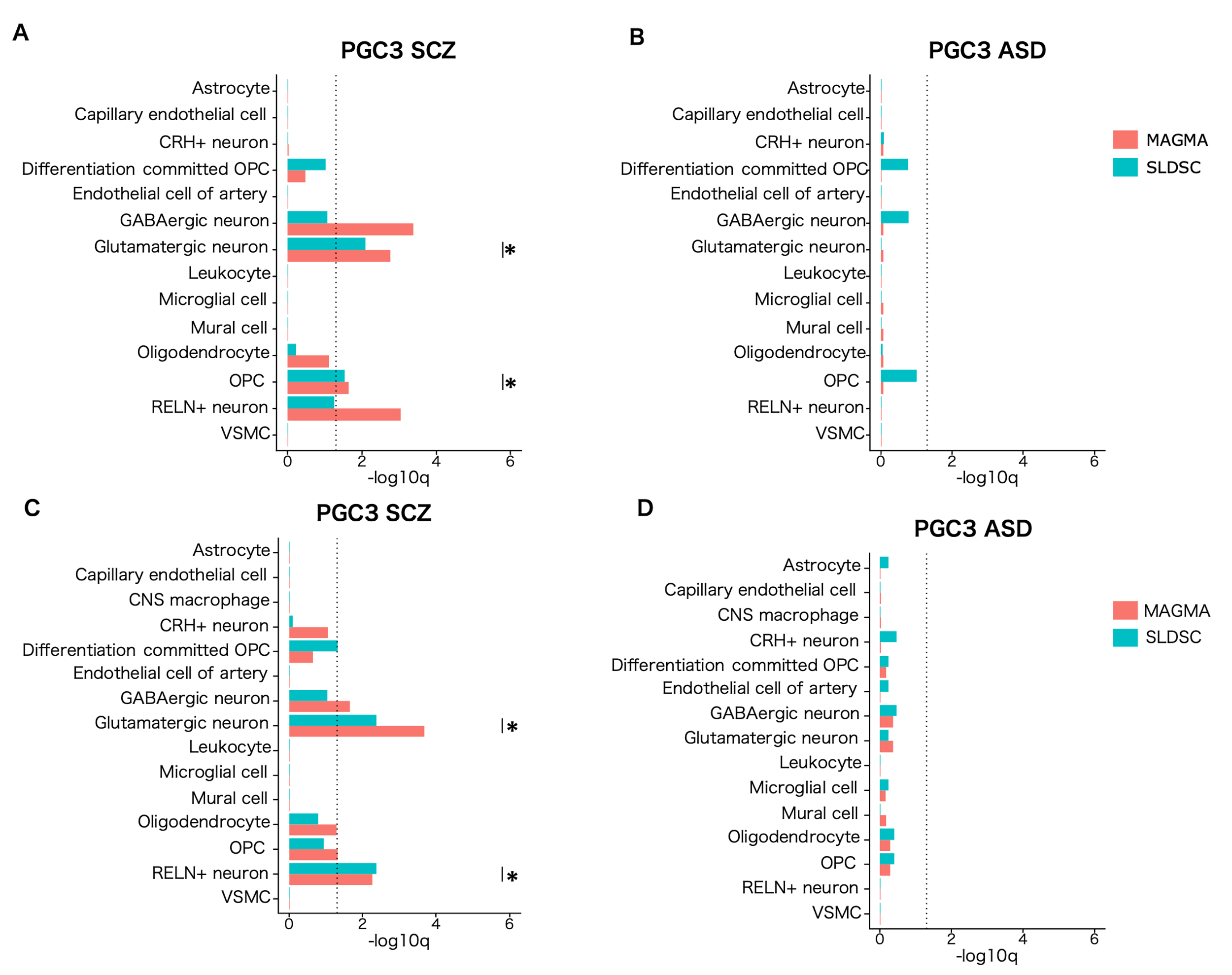


**Supplementary Figure 2.** Cell-type-specific enrichment of common genetic risks for psychiatric disorders in the white matter of BA4 and the cerebellum. (A–C) Cell-type-specific enrichment of common genetic associations for (A) schizophrenia (SCZ) and (B) autism spectrum disorder (ASD) in BA4 white matter calculated for each cell type using MAGMA (red) and SLDSC (blue). (D–F) Cell-type-specific enrichment of common genetic associations for (A) SCZ and (B) ASD, and in cerebellar white matter calculated for each cell type using MAGMA (red) and S-LDSC (blue). Dotted lines indicate significance thresholds (FDR < .05). Asterisks indicate cell populations that satisfied FDR < .05 in both SLDSC and MAGMA analyses.


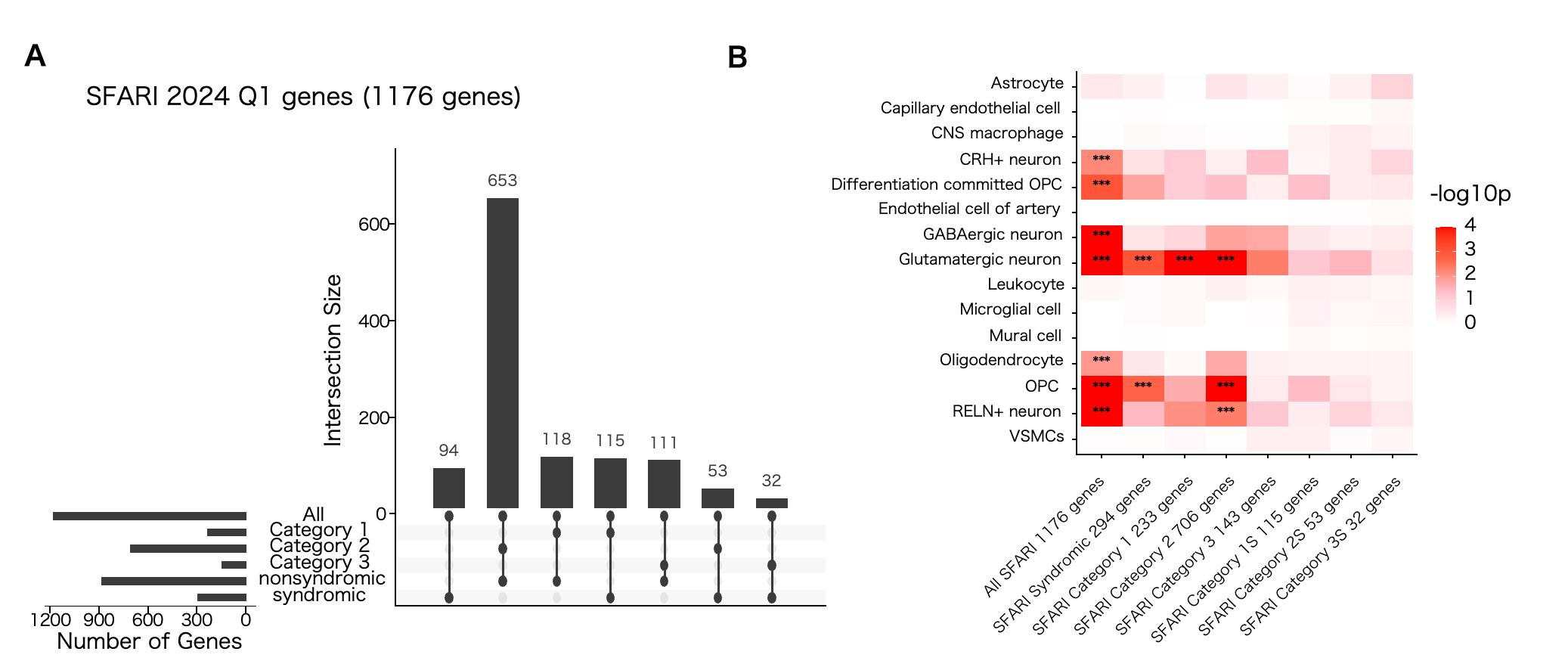


**Supplementary Figure 3.** Enrichment of SFARI genes in the top decile of each specific cell type. (A) Upset plot for gene scoring-based categories in the SFARI database. The number of genes in each category is shown. Syndromic genes are associated with syndromic ASD phenotypes, and categories 1–3 denote the robustness of the evidence, with each signifying high confidence, a strong candidate, and suggestive evidence. (B) Cell-type-specific enrichment of SFARI genes. Cell-type-specific enrichment was calculated using EWCE for each category in the SFARI database. Category #S (# represents 1–3) indicates genes that belong to both category # and syndromic. For plotting and visibility, *P*-values calculated as 0 were replaced by 1e-4. ***FDR < 0.001.


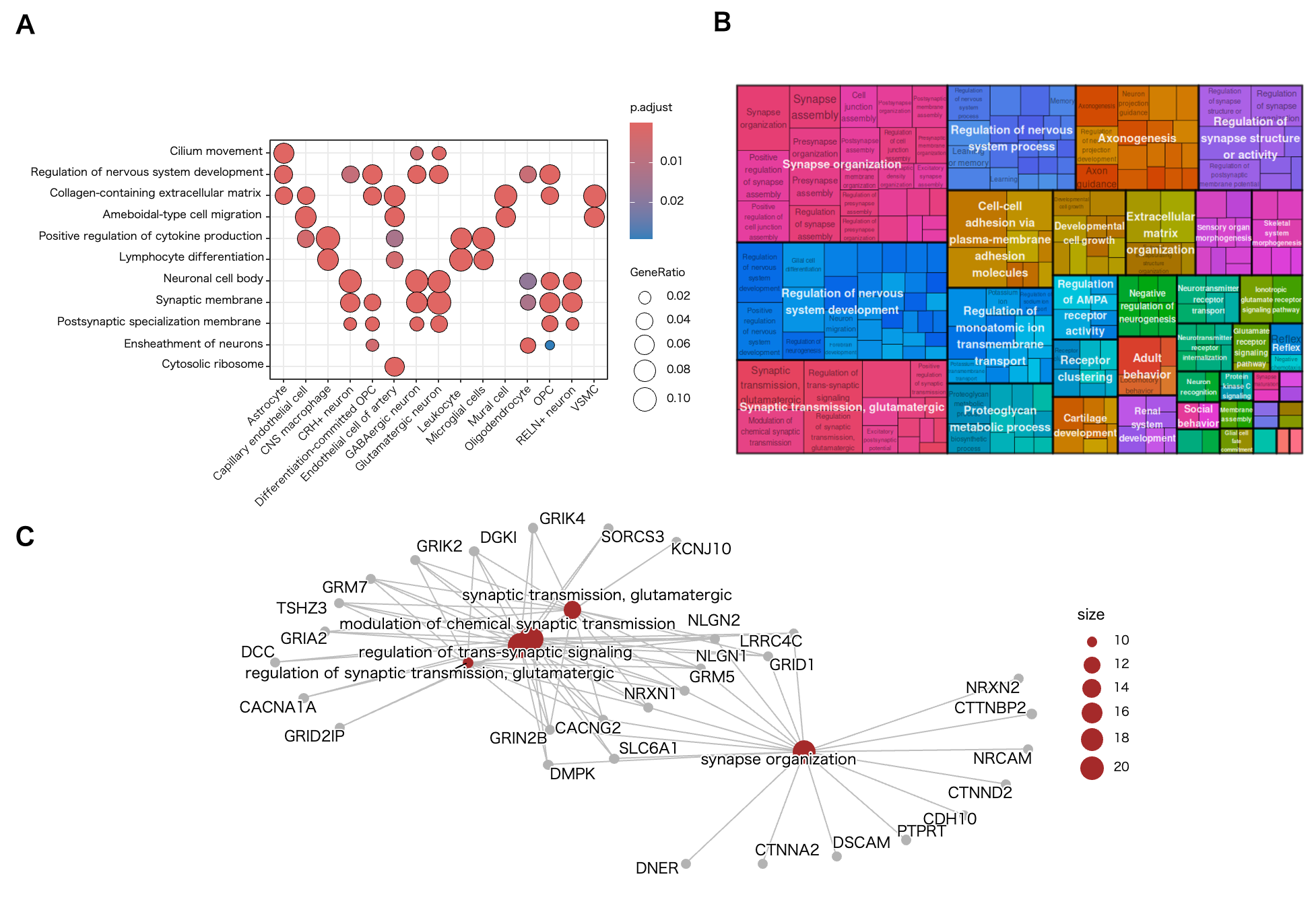


**Supplementary Figure 4.** Convergence of risk genes for SCZ and ASD synaptic genes. (A) Dot plot of GO terms enriched for the top decile of gene expression specificity for each cell type. (B) Space-filling visualization (tree map) of terms grouped by the representative term (GO biological process) for enriched pathways in the top decile of gene expression specificity for oligodendrocyte precursor cells. (C) Gene ontology overrepresentation analysis of 75 genes that are common in the top decile of gene expression specificity for oligodendrocyte precursor cells, SFARI genes, and SCZ MAGMA nominal significance.

**
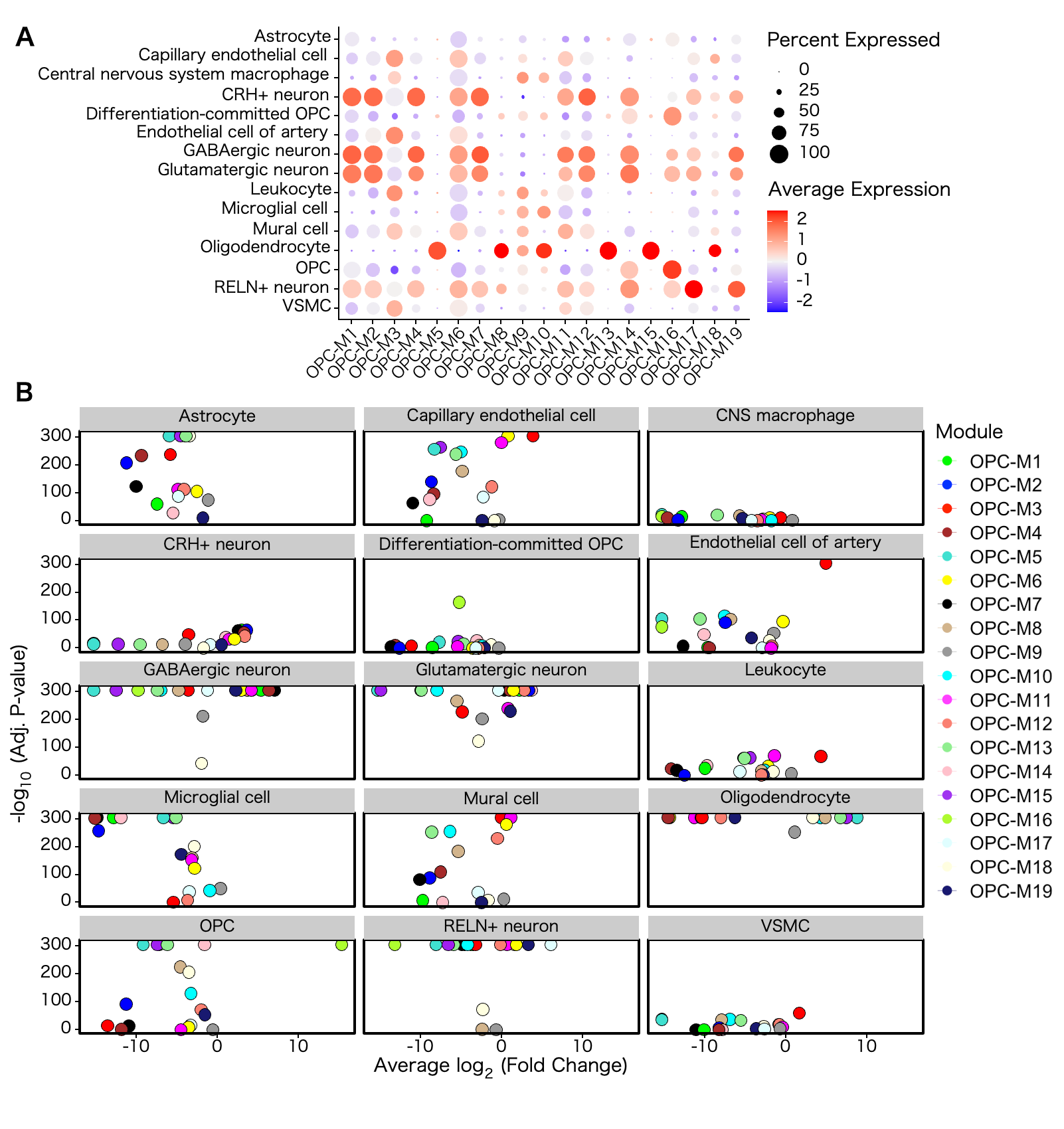
**

**Supplementary Figure 5.** Differential module expression of co-expressing modules across cell populations. (A) Dot plot of the relative expression of each co-expressing module in each cell population. (B) Volcano plot of differential co-expressing module expression in each cell population identified by FindAllDMEs in hdWGCNA. Each colored dot represents the corresponding expressing module. Statistics were performed using the Wilcoxon rank-sum test.


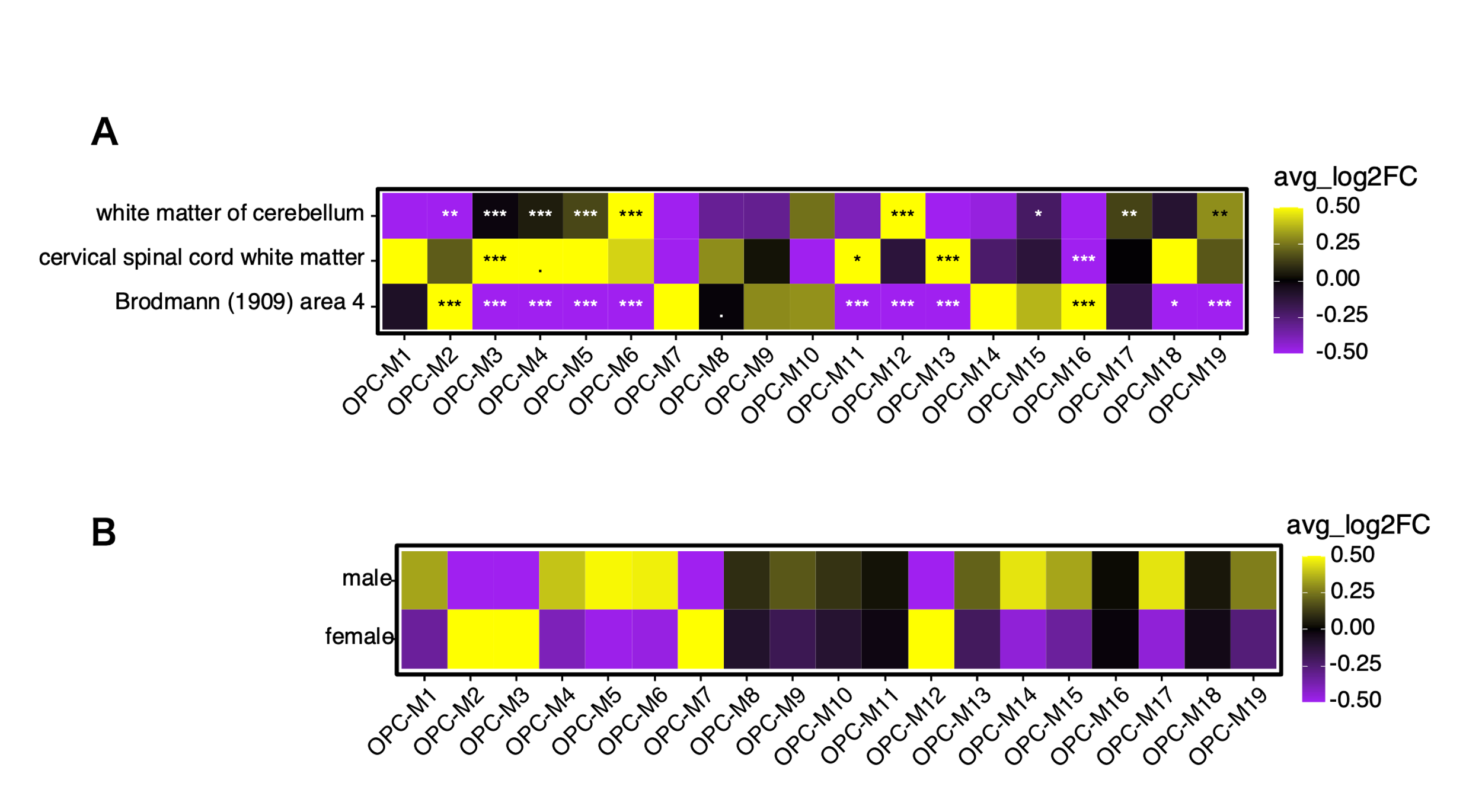


**Supplementary Figure 6.** Differential module expression of co-expressing modules across reginon, sex.

Heatmap of differential module expression of each co-expressing modules in each cell populations. The color shows average expression levels in the condition. The differential expression gene were identified by FindDMEs in hdWGCNA. Statistics were performed by wilcoxon ranksum test. *: FDR<0.05, **: FDR<0.01, ***: FDR<0.001.


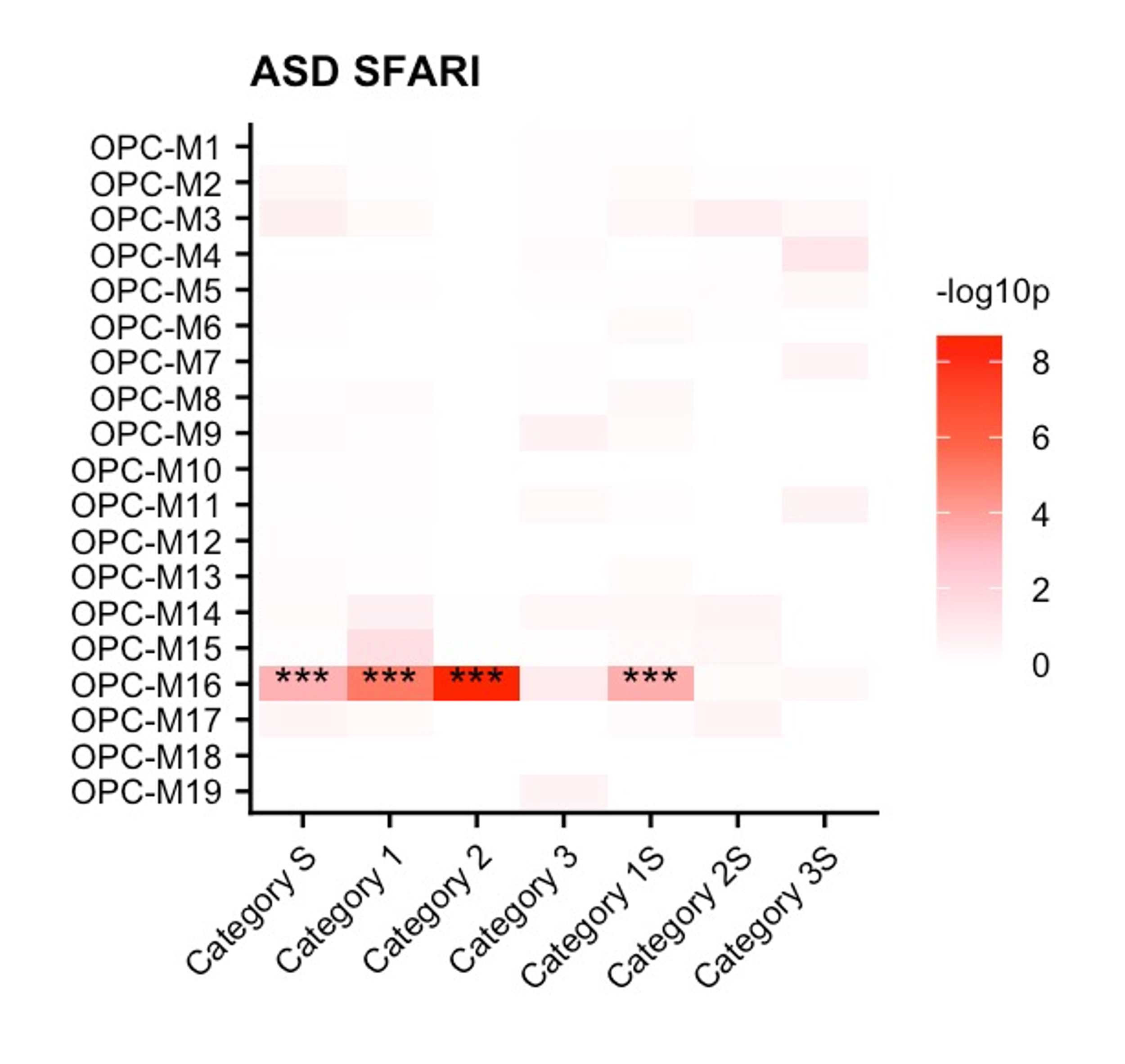


**Supplementary Figure 7.** Heatmap representation of over-representation analysis of each co-expressing module in each ASD susceptibility gene category in SFARI. ***FDR < .001.


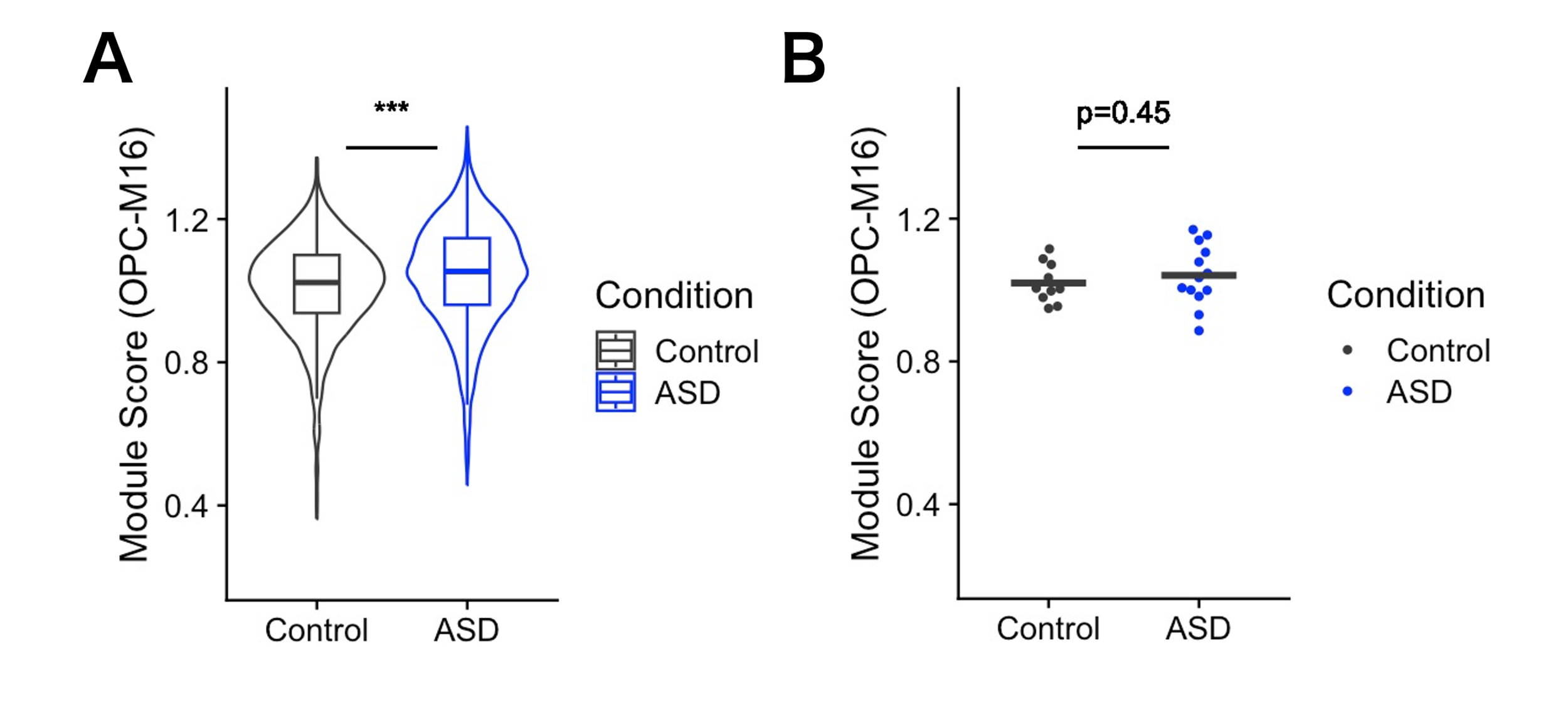


**Supplementary Figure 8.** Expression level of OPC-M16 calculated as a module score in the ASD data set. (A) OPCs from the prefrontal cortex of patients with ASD exhibited significantly elevated OPC-M16 module scores. Statistics were performed using the rank-sum test. Error bars represent the standard error of the mean. (B) Elevation was not significant in patients with ASD when tested by averaging cells from the same patients and after adjusting for covariates. Statistical significance was determined using linear mixed-effects models, accounting for relevant covariates. **P* < 0.05, ****P* < 0.001.


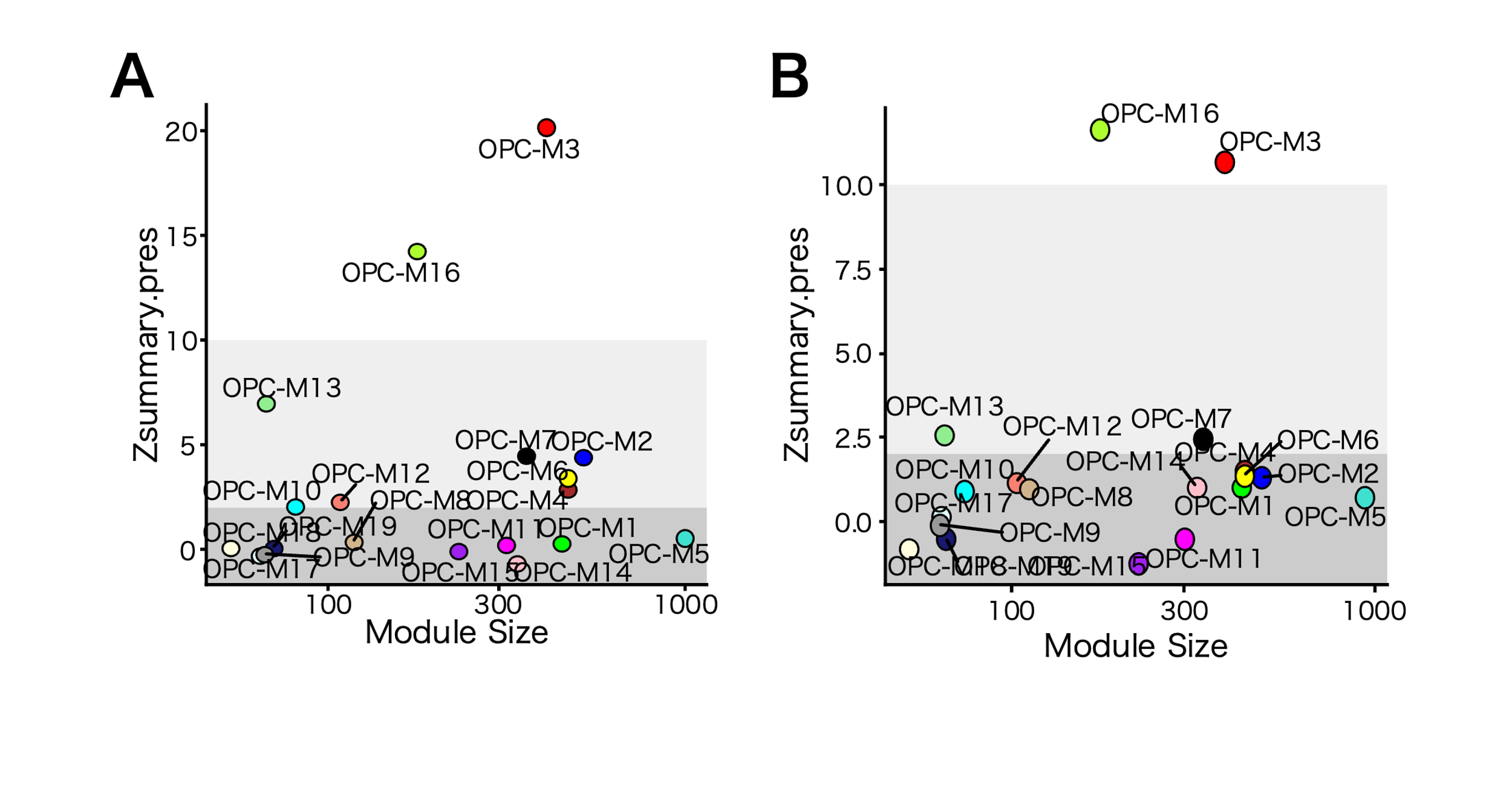


**Supplementary Figure 9.** Module preservation score of each colored dot represents the score for each co-expressing module in the SCZ (A) and ASD data sets (B). The gray area (Z < 2) indicates that the co-expressing module is not preserved, the pale gray area (2 < Z ≤ 10) that the co-expressing module is moderately preserved, and the white area (Z > 10) that the co-expressing module is highly preserved.


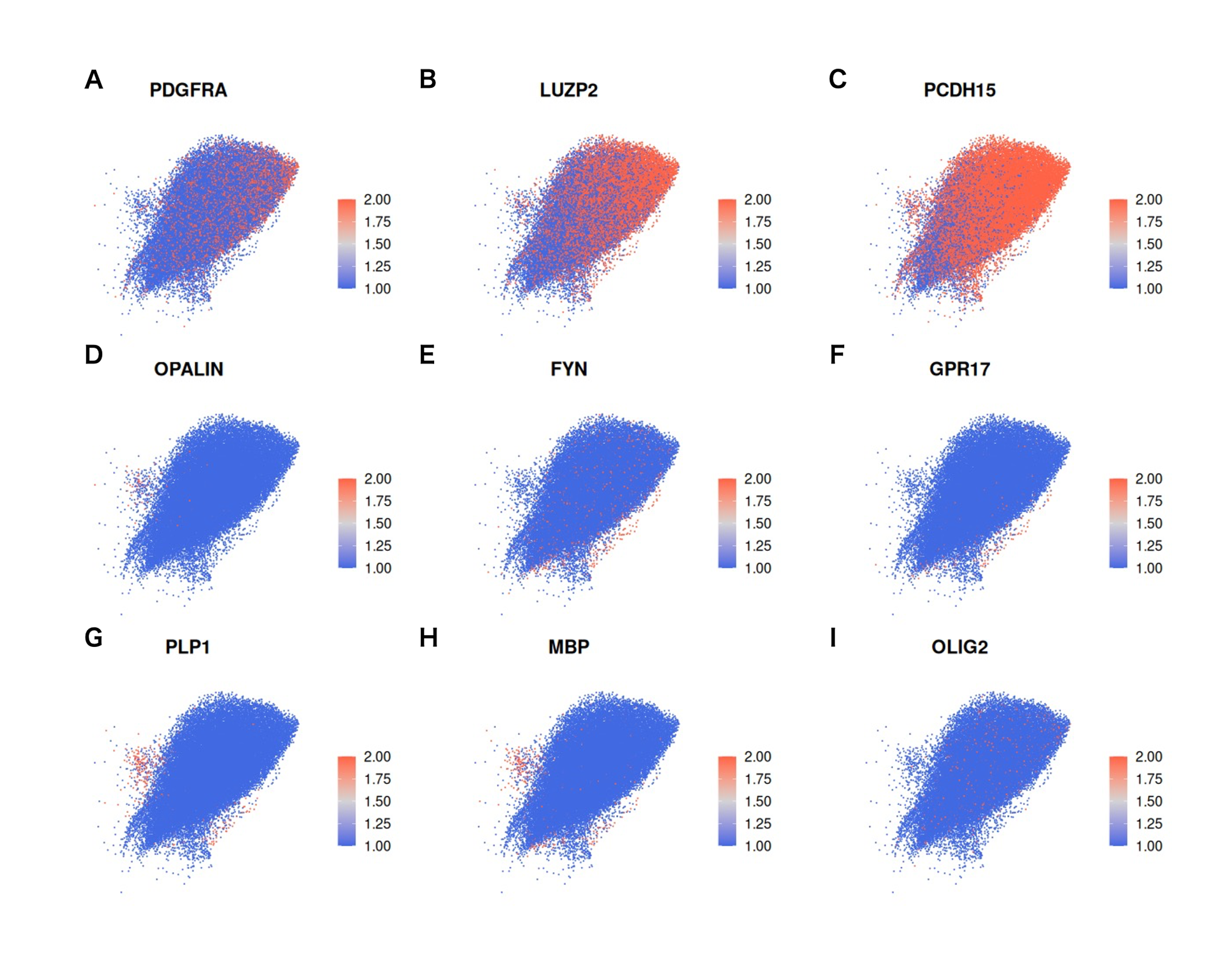


**Supplementary Figure 10.** Feature plot of oligodendrocyte lineage markers using the SCZ data set. Markers are known to be highly expressed within the cell type compared with other cell types in the oligodendrocyte lineage and are not exclusively expressed within the cell type. For visibility, OPCs within 5 SDs of the mean UMAP coordinates are shown (20 cells that are outside were excluded from the plot). (A–C). Markers for OPCs. (D–F) Markers for differentiation-committed OPCs. (G–I) Markers for oligodendrocytes. Expression is shown as scaled values.


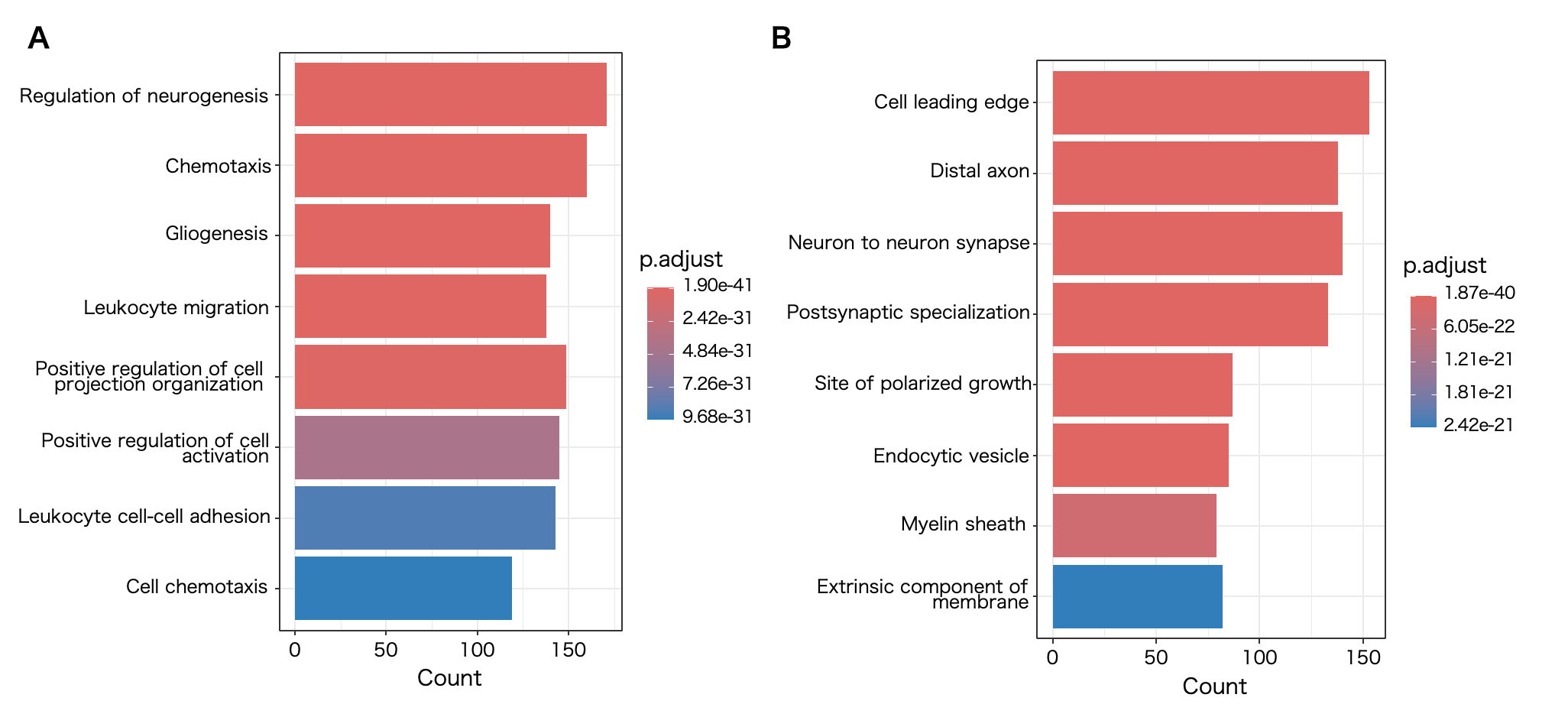


**Supplementary Figure 11.** Bar plot of enriched Gene Ontology (GO) terms in the genes differentially expressed in OPCs expressing DISC1Δ-3 from the mouse. The differentially expressed genes (DEGs) were calculated from the bulk RNA-seq data for OPCs downloaded from the GEO database with accession number [GSE25456](https://www.ncbi.nlm.nih.gov/geo/query/acc.cgi?acc=GSE254569). The calculation was performed using DESeq2 and the significance threshold for DEGs was set at *P*.adjust < 0.01. In total, 3295 genes were identified. (A) GO term for the biological process. (B) GO term for the cellular components.

**Supplementary Table 1.** Top 10 genes in the OPC-M16 co-expressing module.

| **Gene** | **Synapse function** | **OPC function** | **Associated psychiatric disorders** | **Ref** |
| --- | --- | --- | --- | --- |
| **PCDH15** | Synapse organization and cell adhesion in neurons | OPC proliferation and process morphology | BP, ASD, SCZ (CNV) | [^1^](#_ENREF_1)^,^[^2^](#_ENREF_2) |
| **NLGN1** | Postsynaptic protein involved in synapse formation | Expressed in OPCs | ASD, SCZ | [^3^](#_ENREF_3) |
| **LRP1B** | Interact with post-synaptic density protein | Essential for differentiation of OPCs | ALZ, SCZ | [^4-7^](#_ENREF_4) |
| **CSMD1** | Synapse formation | Expressed in OPCs | SCZ, ALZ and ASD | [^8^](#_ENREF_8)^,^[^9^](#_ENREF_9) |
| **OPCML** | Influences synaptic plasticity | Expressed in OPCs | SCZ | [^10^](#_ENREF_10) |
| **LRRC4C** | Involved in neural circuit formation | Expressed in OPCs | SCZ | [^3^](#_ENREF_3)^,^[^11^](#_ENREF_11) |
| **FGF14** | Presynaptic protein that regulates synaptic plasticity | Expressed in OPCs (higher than OLs) | Depression, addiction, SCZ, ALZ | [^12^](#_ENREF_12)^,^[^13^](#_ENREF_13) |
| **LRRTM4** | Post-synaptic organization, synapse formation | Expressed in OPCs | ASD and cognitive function | [^14^](#_ENREF_14) |
| **LSAMP** | Modulates synapse activity, primarily serotonergic | Role in OPC differentiation being explored | Suicidal behavior, depression | [^15-17^](#_ENREF_15) |
| **NRXN1** | Presynaptic adhesion protein essential for synapse | Expressed in OPCs | ASD and cognitive function | [^3^](#_ENREF_3) |
| **MDGA2** | Synapse development | Expressed in OPCs | ASD, SCZ | [^18^](#_ENREF_18) |

The information about expression was obtained from human protein atlas^[19](#_ENREF_19)^. SCZ: schizophrenia, ASD: autism spectrum disorder, ALZ: Alzheimer’s disease, OPC: oligodendrocyte precursor cell.

1. Darwish, M.*, et al.* Neuronal SAM68 differentially regulates alternative last exon splicing and ensures proper synapse development and function. *Journal of Biological Chemistry* **299**(2023).

2. Zhen, Y.*, et al.* Protocadherin 15 suppresses oligodendrocyte progenitor cell proliferation and promotes motility through distinct signalling pathways. *Communications Biology* **5**(2022).

3. Südhof, T.C. Synaptic Neurexin Complexes: A Molecular Code for the Logic of Neural Circuits. in *Cell*, Vol. 171 745-769 (Cell Press, 2017).

4. Soheili-Nezhad, S., van der Linden, R.J., Olde Rikkert, M., Sprooten, E. & Poelmans, G. Long genes are more frequently affected by somatic mutations and show reduced expression in Alzheimer's disease: Implications for disease etiology. *Alzheimers Dement* **17**, 489-499 (2021).

5. Lin, J.P., Mironova, Y.A., Shrager, P. & Giger, R.J. LRP1 regulates peroxisome biogenesis and cholesterol homeostasis in oligodendrocytes and is required for proper CNS myelin development and repair. *Elife* **6**(2017).

6. Tian, Q.B.*, et al.* Interaction of LDL receptor-related protein 4 (LRP4) with postsynaptic scaffold proteins via its C-terminal PDZ domain-binding motif, and its regulation by Ca/calmodulin-dependent protein kinase II. *Eur J Neurosci* **23**, 2864-2876 (2006).

7. Timms, A.E.*, et al.* Support for the N-methyl-D-aspartate receptor hypofunction hypothesis of schizophrenia from exome sequencing in multiplex families. *JAMA Psychiatry* **70**, 582-590 (2013).

8. Hong, S.*, et al.* Complement and microglia mediate early synapse loss in Alzheimer mouse models. *Science* **352**, 712-716 (2016).

9. Ripke, S.*, et al.* Genome-wide association study identifies five new schizophrenia loci. *Nature Genetics* **43**, 969-978 (2011).

10. Zhang, Z.*, et al.* The Schizophrenia Susceptibility Gene OPCML Regulates Spine Maturation and Cognitive Behaviors through Eph-Cofilin Signaling. *Cell Reports* **29**, 49-61.e47 (2019).

11. Choi, Y.*, et al.* NGL-1/LRRC4C deletion moderately suppresses hippocampal excitatory synapse development and function in an input-independent manner. *Frontiers in Molecular Neuroscience* **12**(2019).

12. Di Re, J., Wadsworth, P.A. & Laezza, F. Intracellular Fibroblast Growth Factor 14: Emerging Risk Factor for Brain Disorders. *Front Cell Neurosci* **11**, 103 (2017).

13. Yan, H., Pablo, J.L. & Pitt, G.S. FGF14 regulates presynaptic Ca2+ channels and synaptic transmission. *Cell Rep* **4**, 66-75 (2013).

14. DeWit, J.*, et al.* Unbiased discovery of Glypican as a receptor for LRRTM4 in regulating excitatory synapse development. *Neuron* **79**, 696-711 (2013).

15. Bregin, A.*, et al.* Expression and impact of Lsamp neural adhesion molecule in the serotonergic neurotransmission system. *Pharmacology Biochemistry and Behavior* **198**(2020).

16. Koido, K.*, et al.* Associations between LSAMP gene polymorphisms and major depressive disorder and panic disorder. *Translational Psychiatry* **2**(2012).

17. Sharma, K.*, et al.* Cell type- and brain region-resolved mouse brain proteome. *Nature Neuroscience* **18**, 1819-1831 (2015).

18. Zhuo, C.*, et al.* Associations of cognitive impairment in patients with schizophrenia with genetic features and with schizophrenia-related structural and functional brain changes. *Front Genet* **13**, 880027 (2022).

19. Karlsson, M.*, et al.* A single-cell type transcriptomics map of human tissues. *Sci Adv* **7**(2021).
